# Primary and secondary prevention interventions for cardiovascular disease in low-income and middle-income countries: a systematic review of economic evaluations

**DOI:** 10.1186/s12962-018-0108-9

**Published:** 2018-06-14

**Authors:** Leopold Ndemnge Aminde, Noah Fongwen Takah, Belen Zapata-Diomedi, J. Lennert Veerman

**Affiliations:** 1 Faculty of Medicine, School of Public Health, The University of Queensland, Brisbane, QLD 4006 Australia; 2 Non-communicable Diseases Unit, Clinical Research Education, Network & Consultancy, Douala, Cameroon; 3 London School of Hygiene and Tropical Medicine, London, UK; 4 School of Medicine, Griffith University, Gold Coast, QLD 4222 Australia; 5 Cancer Research Division, Cancer Council NSW, Woolloomooloo, NSW 2011 Australia

**Keywords:** Prevention, Cardiovascular disease, Primary, Secondary, Cost-effectiveness, LMICs

## Abstract

**Background:**

Cardiovascular disease (CVD) is the leading cause of deaths globally, with greatest premature mortality in the low- and middle-income countries (LMIC). Many of these countries, especially in sub-Saharan Africa, have significant budget constraints. The need for current evidence on which interventions offer good value for money to stem this CVD epidemic motivates this study.

**Methods:**

In this systematic review, we included studies reporting full economic evaluations of individual and population-based interventions (pharmacologic and non-pharmacologic), for primary and secondary prevention of CVD among adults in LMIC. Several medical (PubMed, EMBASE, SCOPUS, Web of Science) and economic (EconLit, NHS EED) databases and grey literature were searched. Screening of studies and data extraction was done independently by two reviewers. Drummond’s checklist and the National Institute for Health and Care Excellence quality rating scale were used in the quality appraisal for all studies used to inform this evidence synthesis.

**Results:**

From a pool of 4059 records, 94 full texts were read and 50 studies, which met our inclusion criteria, were retained for our narrative synthesis. Most of the studies were from middle-income countries and predominantly of high quality. The majority were modelled evaluations, and there was significant heterogeneity in methods. Primary prevention studies dominated secondary prevention. Most of the economic evaluations were performed for pharmacological interventions focusing on blood pressure, cholesterol lowering and antiplatelet aggregants. The greatest majority were cost-effective. Compared to individual-based interventions, population-based interventions were few and mostly targeted reduction in sodium intake and tobacco control strategies. These were very cost-effective with many being cost-saving.

**Conclusions:**

This evidence synthesis provides a contemporary update on interventions that offer good value for money in LMICs. Population-based interventions especially those targeting reduction in salt intake and tobacco control are very cost-effective in LMICs with potential to generate economic gains that can be reinvested to improve health and/or other sectors. While this evidence is relevant for policy across these regions, decision makers should additionally take into account other multi-sectoral perspectives, including considerations in budget impact, fairness, affordability and implementation while setting priorities for resource allocation.

**Electronic supplementary material:**

The online version of this article (10.1186/s12962-018-0108-9) contains supplementary material, which is available to authorized users.

## Background

Cardiovascular disease (CVD) is the number one cause of mortality globally, accounting for about 31% of worldwide deaths. Estimates from the Global Burden of Disease (GBD) 2015 study showed that there were 422 million cases of CVD globally, and deaths from CVD have increased from 12.6 million in 1990 to 17.9 million in 2015 [1]. Over four-fifths of premature mortality (deaths before age 70 years) from non-communicable disease (NCD) occurs in low-income and middle-income countries (LMICs), and over a third is caused by CVD [2]. While the trend (1990–2015) in age-standardized prevalence of CVD is declining in high-income countries (HIC), this is not very obvious for most LMICs, where current rates are > 9000 prevalent cases per 100,000 persons. Likewise, there have been significant declining trends in age-standardized CVD mortality rates in all HICs, however similar changes have not been observed for the majority of sub-Saharan Africa and Southeast Asia [1].

The epidemiologic transition and demographic changes (population growth and ageing) have contributed to the CVD burden in LMICs. Evidence from research on early life (in utero) exposures, genes, and the environment have added to the understanding of the development and occurrence of CVDs in adulthood. Furthermore, metabolic (high blood pressure, high blood glucose, dyslipidemias, obesity) and behavioural (tobacco use, unhealthy diet, physical inactivity) risk factors are time-honored drivers fueling this CVD epidemic around the world [3]. A number of these risk factors are modifiable, and are targeted to curtail this burden via preventive and or treatment strategies.

There are several models of prevention, including population-wide and individual approaches targeting either individual risk factors, or multiple risk factors [4]. These strategies may be geared towards individuals with risk factors to prevent incidence of CVD events like cerebrovascular accidents and ischaemic heart disease (primary prevention) or in those with CVD events to prevent recurrence (secondary prevention) or reduce long-term impairment and disability resulting from a CVD event (tertiary prevention) [4]. Preventive interventions include (but are not limited to) medical procedures, pharmacological (blood pressure and cholesterol lowering medication, anti-platelet aggregants, thrombolytic agents) and non-pharmacological (health education, taxation, legislation) interventions.

Recognizing the plethora of individual country healthcare needs, and ever limited resources, the requisite for economic evaluation of interventions has been increasingly acknowledged [5]. This economic evidence forms one of the parameters for government and health policy makers as they decide on where to invest [6].

While there is overwhelming evidence in HICs from economic evaluations on the cost-effectiveness of interventions for CVD prevention, this is not the case for LMICs. Moreover, the transferability and implementation of interventions trailed in HIC to LMICs is debatable [7]. Among others, there are differences in effectiveness and cost related to variations in socio-cultural, environmental, demographic, disease profiles and importantly, human and financial resources. Especially in Africa, LMICs are not only faced with the growing NCD burden, but are also afflicted by still-large burdens of infectious disease, nutritional disorders, neonatal and maternal mortality [8]. Thus, considering the inherent limited financial resources amidst these colossal health needs (communicable and non-communicable), their governments are faced with a greater challenge in choosing interventions that offer good value for money.

Based on the above, there is great need for robust evidence on which interventions are cost-effective to inform policy decisions. We must acknowledge that this is not the first review on economic evaluations for CVD. Suhrcke et al. [7] and Shroufi et al. [9] have previously reviewed the topic, though their work included studies only up to 2009 and 2010, respectively. The study by Suhrcke and colleagues had a number of limitations. While they used a reasonably sensitive database search strategy, they did not assess grey literature and so it is likely that they might have missed some important studies. Also, their quality assessment was based on authors’ statements on methods, instead of objective quality assessment tools. Furthermore, it is unclear why the study by Shroufi et al. included few studies. However, we noticed that in terms of geography, they used continental or regional names in their search. Including specific country names would likely have increased the sensitivity of their search strategy in capturing more studies.

Considering the time since the conduct of these studies and the above shortcomings, there is a clear need to provide updated and contemporary evidence of interventions providing the most health gains with minimal costs, in the prevention CVD in LMICs.

## Methods

This systematic review has been reported in accordance with the Preferred Reporting Items for Systematic Reviews and Meta-analyses (PRISMA) guidelines [10], (Additional file 1). Our review was registered in the PROSPERO International prospective register of systematic reviews (registration number: CRD42016043510) at the Centre for Reviews and Dissemination, University of York, UK and the protocol has been published [11].

### Objective

The objective of this study was to identify, via a comprehensive synthesis, those interventions that are cost-effective in the prevention of cardiovascular diseases in low-income and middle-income countries in order to inform and guide health policy in these countries in curbing the growing CVD burden.

### Criteria for eligibility

For inclusion in this review, studies had to be primary (observational studies and randomized control trials) or modelling studies reporting on interventions for primary or secondary prevention of CVD among adults (> 18 years) from LMICs. Only those reporting full economic evaluations (cost-effectiveness analysis (CEA), cost-utility analysis (CUA) or cost–benefit analysis (CBA)) with clear identification of comparators (either current practice or the ‘do nothing’ scenario) and outcome measures such as cost per life year gained or per unit clinical outcome, cost per quality adjusted life year (QALY) or cost per disability adjusted life year (DALY) were considered. All studies written in English or French were included. We excluded narrative reviews, letters to the editor, case series with sample size less than 50 participants, and others lacking explicit information on methods.

### Data sources and search strategy

We conducted a comprehensive search of several medical and economic literature databases from inception to 10 July 2017 (date of last search). Databases searched were: MEDLINE via PubMed, EMBASE, SCOPUS, Web of Science, EconLit (American Economic Association), NHS Economic Evaluation Database (NHS EED) and Database of Abstracts of Reviews of Effects (DARE) via Centre for Reviews and Dissemination (CRD) database. The WHO AFROLIB, African Journals Online (AJOL) and Africa Index Medicus were also searched for literature specific to Africa. Additional file 2 shows in detail the search strategy which was adapted for each of the searched databases.

For grey literature, we searched websites of research organizations such as Disease Control Priorities (DCP) and WHO-CHOICE. We also searched Google Scholar and where necessary, corresponding authors were contacted via email.

To further complement our database search, we perused the reference lists of the previous review studies and articles that met our inclusion criteria.

### Screening and data extraction

Two reviewers independently screened titles/abstracts (LNA and BZ-D), independently screened full texts and extracted data (LNA and NTF) for studies included in the review. Any disagreements or conflicts were resolved by consensus or consultation with third reviewer (JLV).

Using a preconceived data-extraction form, all relevant data was obtained including first author name and year of publication, study setting, geographic region, country income level (according to 2017 World Bank classification) [12], study design, intervention type and measure, intervention target, risk factor(s) examined, effect estimate (relative risk or effectiveness measure), type of economic evaluation, comparator, outcome, type of sensitivity analysis, economic perspective, incremental cost-effectiveness ratio (ICER), cost-effectiveness as described by authors and the criteria, funding sources. For modelling studies, the type of modelling strategy (micro- or macro-simulation), time horizon and discount rate were recorded while for primary studies, the specific study design, sample size of intervention and control groups, mean age of participants, percentage of male or female participants and length of follow-up data were obtained.

### Quality assessment and appraisal

The reporting and methodological quality of all included studies was independently assessed by two reviewers (LNA and NFT) using the Drummond checklist for economic evaluation studies [13]. This checklist has 35 questions in total distributed under three major sections covering aspects of study design; sources and quality of data collected; data analysis and interpretation of results. These questions have *Yes, No, Not clear* and *Not applicable* as possible responses (see Additional file 3). We then used the NICE scale in rating quality, with ‘++’ for good quality, ‘+’ for moderate quality, and ‘−’ for poor quality studies denoting low, moderate and high risk of bias, respectively [14]. The quality assessment was for overall study level and not the outcomes for included studies. Discrepancies in quality assessment were resolved by consensus.

### Data management and synthesis

This has been previously described in the review protocol [11]. Briefly, EndNote V.7.4 software was used for removal of duplicate records. The remaining studies uploaded into Rayyan QCRI [15], which is a web and mobile-app internet-based program that assists collaboration between reviewers through the screening and selection process. All data extracted from final included studies were entered to Microsoft Excel 2013 spreadsheet. Data synthesis involved stratifying and summarizing the evidence by preventive intervention type, appraising the economic evaluation methods used for assessing interventions and presentation of cost-effectiveness outcomes. Inter-rater reliability for study inclusion and quality assessment was assessed using Cohen’s kappa coefficient (k). All analyses were done using STATA v. 15 (STATA corp, Texas, USA).

## Results

### Review search results

**Fig. 1:**
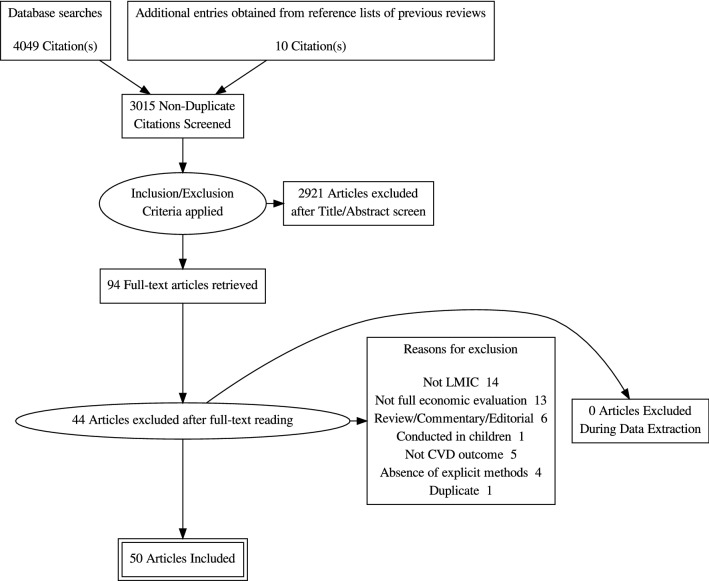
PRISMA flow diagram

The database search yielded 4049 entries, and ten additional studies were obtained from the reference lists of prior reviews [7, 9] giving a total of 4059 studies. After removal of duplicates, 3016 studies were left. The titles and abstracts of these studies were screened independently by two reviewers (LNA and BZ-D) for relevance. After exclusion of clearly irrelevant articles, 94 potentially eligible articles remained which were then read in detail independently by two reviewers (LNA and NFT). Of these, 50 met our inclusion criteria. Data extraction and quality assessment was done by two independent reviewers (LNA and NFT). Inter-rater reliability (Kappa statistic) for study inclusion was high (k = 0.89). Figure 1 shows the PRISMA flow diagram of the study selection process.

### General characteristics of included studies

**Fig. 2:**
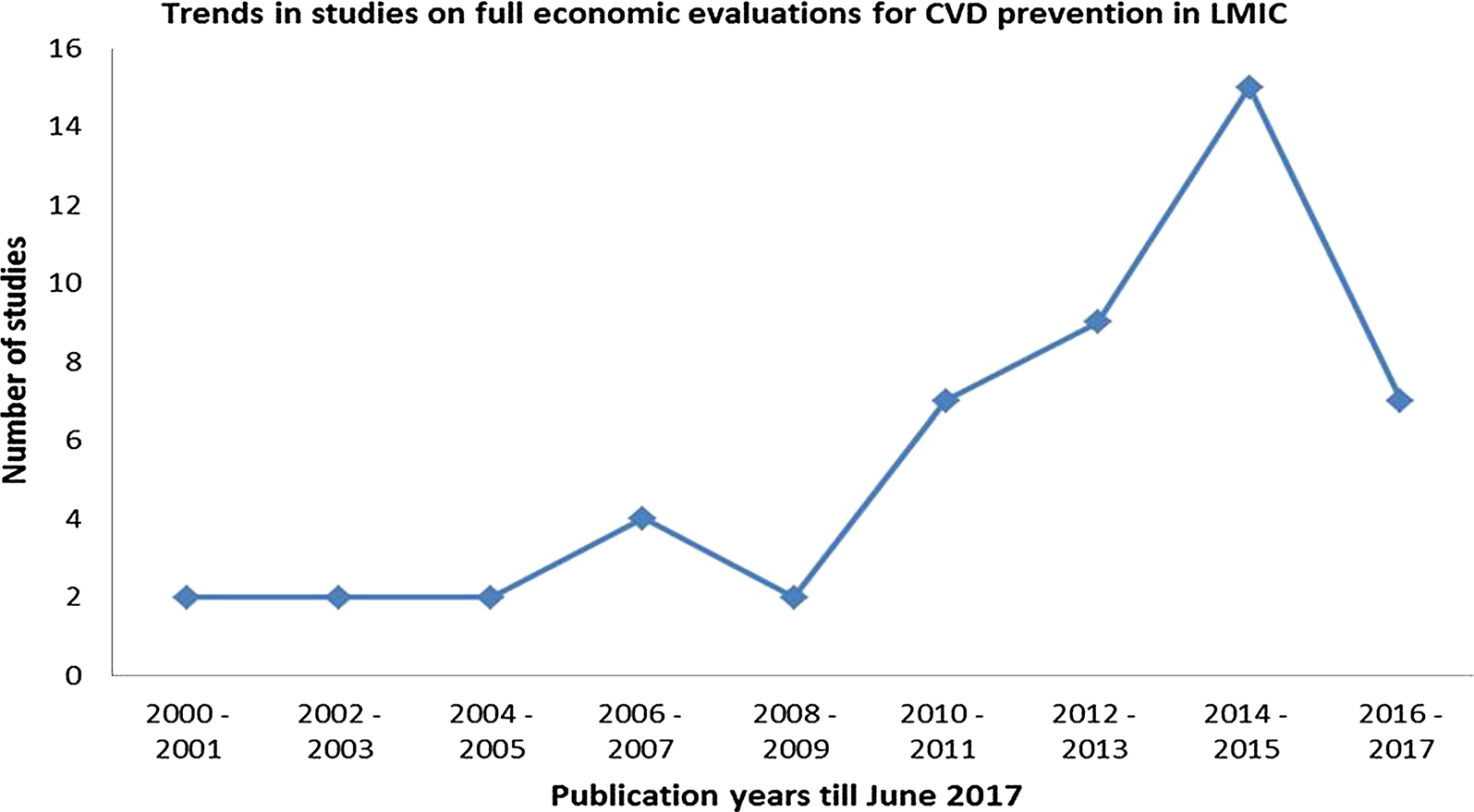
Publication trends by year

Included studies were published from the year 2000, with numbers progressively increasing (Fig. 2). Forty-four (88%) of these studies were from single countries, and six (12%) conducted for two or more countries. Most included studies were conducted for East Asia and the Pacific (n = 16, 32%), Latin America and the Caribbeans (n = 10, 20%), and sub-Saharan Africa (n = 8, 16%), six (12%) studies where from multiple regions. The majority of studies were conducted for upper middle (n = 31, 62%) and lower middle (n = 10, 20%) income countries. Only three studies were conducted in low-income countries [16–18].

In 22 (44%) studies, the main focus was primary prevention while 18 (36%) were on secondary prevention. Four were economic evaluations of hypertension or CVD screening [19–22]. The majority (n = 32, 64%) of studies were pharmacological interventions, followed by a mix of health education/promotion, legislative and medical procedure interventions. Most interventions targeted individuals (n = 38, 76%) with only seven for population-based interventions [20, 22–27], and five studies including both individual and population-based strategies [28–32]. Among CVD risk factors, twenty studies looked at interventions for single risk factors, on high blood pressure (BP) alone (n = 13, 38.2%), followed by high cholesterol (n = 3, 8.8%), atrial fibrillation (n = 2, 5.9%), salt (n = 1, 2.9%) and tobacco (n = 1, 2.9%). Fourteen (41.1%) studies assessed multiple risk factors including varied combinations of BP, cholesterol, smoking and salt intake; 13 of which assessed absolute CVD risk [16–18, 26, 28–36], with one comparing CVD risk in those with and without diabetes [17]. Two (5.9%) studies were in persons with atrial fibrillation [37, 38]. Details of study characteristics are shown in Table 1.

**Table 1:** Included studies with interventions, intervention types and targets, effectiveness estimates, outcome and conclusions

Author, pub year	Country	Region	Intervention	Intervention type	Target	Intervention effect/effectiveness estimate	Source of effect	Outcome: incremental cost effectiveness ratio (ICER)	Conclusion	Currency and year (used in analysis)
Akkazieva et al. 2009	Kyrgyzstan	2	Assessed CE of several primary and secondary interventions to prevent and control CVD	Primary + secondary	Population	Health education through mass media to reduce cholesterol: reduction in total cholesterol = 2%; health education through mass media to reduce hypertension: difference between actual SBP and 115 mmHg = − 2%; hypertension lowering drug treatment and education on dietary change: difference between actual SBP and 115 mmHg = − 33%. Cholesterol lowering drug and life style modification: reduction in total cholesterol = 20%. Combination of drug therapy for at risk patients: reduction in absolute CVD risk = 20%. Opportunistic screening and counselling for CVD risk factor: Difference between actual SBP and 115 mmHg = − 2%	Meta-analyses of RCTs	Highly CE:: Diuretics (for HF) = 1115/DALY, [Diu + ACEi + Exercise] = 1567/DALY, Mass media cholesterol = 3822/DALY, BB (for HF) = 3915/DALY, Aspirin (post acute IHD) = 4179/DALY, Mass media salt campaign = 6203/DALY, HTN treatment (> 160 mmHg) = 7615/DALY, Aspirin (post acute stroke) = 7757, ACEi (post acute IHD) = 8833/DALY, ACEi (for HF) = 8833/DALY, Aspirin (acute MI) = 11,417/DALY, Aspirin + Anticoagulant = 12,308/DALY//CE:: Mass media smoking = 24,202/DALY, ACEi + Diu (post stroke) = 27,832/DALY, HTN treatment(> 140 mmHg) = 28,863/DALY, [Aspirin + BB + ACEi + Streptokinase](acute MI) = 31,628/DALY, ACEi (acute MI) = 39,504/DALY	Highly CE and CE, some were also not CE. Absolute CVD risk at all thresholds, statin treatment, streptokinase, primary PTCA, individual cholesterol treatment (> 5.7 mmol/L and > 6.2 mmol/L) were all not cost-effective	Kyrgygstan Som, 2005
Amirsadri and Hassani, 2015	Iran	4	Compared CE of treatment with 10 mg Simvastatin in 45 year old men with average (15%) 10 year CVD risk versus no treatment	Primary	Individual	RR for simvastatin for healthy to non-fatal MI = 0.752, healthy to fatal MI = 0.813	Systematic review	US $1113/QALY and US $935/LYG	Highly cost-effective	US dollar, 2014
Amirsadri and Sedighi, 2017	Iran	4	Assessed the CE of Aspirin in primary prevention of MI in men > 45 years with moderate CVD risk of 15% over 10 years versus no treatment	Primary	Individual	For Aspirin: RR of health to non-fatal MI = 0.68, RR of health to fatal MI = 0.87, RR of post MI to non-fatal MI = 0.72, RR of post MI to fatal MI = 0.85, RR of MI to non-fatal MI = 0.44, RR of MI to fatal MI = 0.78	Meta-analyses of RCTs	$864/QALY and $782/LYG	Highly cost-effective	US dollar, 2015
Anderson et al. 2000	South Africa	7	Compared C-E of various ARBs (Candesartan, Valsartan, Irbesartan and Losartan) in reducing sitting DBP	Primary	Individual	Mean reduction in SDBP: Candesartan = 10.57 (9.60–11.54), Valsartan = 7.11 (6.13–8.08), Irbesartan = 9.07 (8.26–9.87)	Meta-analysis	22.34R/mmHg reduction in sDBP for Candesartan, 32.86R/mmHg for Valsartan, 29.65R/mmHg for Irbesartan	Candesartan was most cost-effective for treating HTN	Rands
Anderson et al. 2000	South Africa	7	Administering Ramipril for treatment in post-MI patients with heart failure compared to standard therapy (no Ramipril)	Secondary	Individual	RRR of 27% (11–40%) of all-cause mortality	Single RCT	R16, 808/LYG; For < 65 years = R21, 382/QALY and those > 65 years = R18, 029/QALY	Cost effective	Rands, 1999
Araujo et al. 2007	Brazil	3	Assessed CE of Rosuvastatin vs. Atorvastatin in lowering cholesterol and avoiding CVE	Primary	Individual	Efficacy of Rosuvastatin 43% vs. 37% atorvastatin and every 1 mg/dL drop in LDL-C = CVE RRR of 0.16% (1st year), 0.72% (2nd year), 1.03% (3rd year), 0.90% (4th year), 0.85% (5th year)	Meta-analyses of RCTs	Avoided CVE = Dominant, LYG = Dominant at both LDL thresholds of 160 and 190 mg/dL	Cost effective	Brazilian Reais (R$) in 2007
Araujo et al. 2008	Brazil	3	Assessed CE of prehospital thrombolysis in AMI compared to in-hospital thrombolysis on mortality	Secondary	Individual	OR = 0.83 (0.70–0.98) for reduction in mortality	Meta-analysis	Dominant at 1 and 20 years	Cost effective	Brazilian Reais (R$) in 2005
Basu et al. 2016	China and India	1, 6	Compared 3 alternative BP treatment strategies (treatment to target (TTT), benefit-based tailored treatment (BTT) and hybrid strategy)	Primary	Individual	RR = 2^αx(β1γ^2 + β2γ + β3), where α = postTrt-preTrt BP, β1 for MI = − 1.1009 × 10^−5 and β1 for stroke = − 2.5946 × 10^−5, β2 for MI = 8.6305 × 10^−4 and β2 for stroke = 2.3052 × 10^−3, β3 for MI = 3.5176 × 10^−2, β3 for stroke = 2.2168 × 10^−2, γ = age in years	Meta-analysis of RCTs	US$205-$272/DALY averted for BTT	BTT was cost-effective than TTT or hybrid strategy	US dollar, 2015
Basu et al. 2015	India	1	Assessed the CE of government provided coverage of primary prevention, secondary prevention and tertiary treatment for CVD compared to status quo of no coverage	Primary + secondary	Individual	Primary prevention: [ACEi + CCB]-RR for MI = 0.60–0.71, RR stroke = 0.45–0.58; [Statin]-RR for MI = 0.55–0.74, RR stroke = 0.78–1.00 || Secondary treatment: [Aspirin]-RR for MI = 0.60–0.72, RR for stroke = 0.72–0.84, RR for death = 0.81–0.89; [Beta Blocker]-RR for MI = 0.73–0.87, RR for stroke = 0.68–0.74, RR for death = 0.68–0.85; [ACEi]-RR for MI = 0.70–0.90, RR stroke = 0.56–0.84, RR for death = 0.75–0.95; [Statin]-RR for MI = 0.62–0.82, RR stroke = 0.66–1.00, RR death = 0.69–0.87	Meta-analyses of RCTs	Primary prevention only = $469/DALY, Secondary prevention only = $2404/DALY, Primary plus secondary = $2431/DALY	Primary prevention was most CE	US dollar 2014
Bautista et al. 2013	Argentina, Colombia, Costa Rica, Dominican Republic, Peru, Venezuela	3	Compared benefits of administering polypill containing 3 antiHTNsive (thiazide, atenolol, Ramipril), a statin and aspirin to different high risk groups in Latin America compared to no polypill.	Primary	Individual	RR for fatal vs. nonfatal event: WOMEN (≥ 55 year = 0.85 vs. 0.85, Obese = 0.94 vs. 0.94, WHO abdominal Obesity = 0.87 vs. 0.88, LASO abdominal obesity = 0.91 vs. 0.91, MetS = 0.90 vs. 0.91, High risk = 0.84 vs. 0.85); MEN(0.95 vs. 0.95, 0.95 vs. 0.95, 0.94 vs. 0.94, 0.87 vs. 0.88, 0.95 vs. 0.95, 0.81 vs. 0.79)	Longitudinal study	Women = $268/QALY in high risk group, Men = $449/QALY for age ≥ 55 years; If polypill was used in people with ≥ 15% risk of CVD-implying treatment of 26% of population at $34–$36/QALY	Cost effective	Dollar ($) but year not mentioned
Choosakulchart et al. 2013	Thailand	1	Compared the CE of 3 interventions (Influenza vaccine in all IHD groups, in angina patients only, and in cardiac arrest/MI patients only) versus no influenza vaccination	Secondary	Individual	RR of death in influenza vaccine vs. no vaccine = 0.39, RR of AMI in influenza vaccine vs. no vaccine = 0.85	Cochrane systematic review	Influenza vaccine to Angina patients only was most cost effective (8,240 THB/QALY). However, vaccination to all CHD groups though less cost-effective (33,813 THB/QALY) is recommended as it falls below willingness to pay threshold (100,000 THB/QALY)	Cost-effective	Thai baht 2010
Davies et al. 2013	Turkey	2	Compared the CE of Prasugrel in patients with ACS overall and specific groups (UA-NSTEMI, STEMI, Diabetes, Core cohort) undergoing PCI versus Clopidogrel	Secondary	Individual	RR for all-cause mortality [UA/NSTEMI = 1.55 (1.31–1.84), STEMI = 1.84 (1.52–2.20), recurrent NSTEMI = 2.93 (2.34–3.66), recurrent STEMI = 3.48 (2.77–4.37), stroke = 2.39 (1.44–3.97)	RCT and Prospective cohort	Licensed population = €7294/QALY, UA-NSTEMI = €9371/QALY, STEMI = €4552/QALY, Diabetes = €3036/QALY, Core cohort = €7207/QALY	Cost effective	Euros 2011
Donaldson et al. 2011	India	6	Compare C-E of complete smoking ban versus partial smoking ban (India’s 2008 Prohibition of Smoking in Public Places Rules).	Primary	Population	Complete smoking ban = reduce smoking by 3.4% & exposure to SHS by 86%; Partial smoking ban = reduce exposure to SHS by 22% but no change on smoking prevalence.	Observational studies	Without medical treatment = US $9.13 (2.24–112)/LYG and US $229 (37–387)/acute MI case averted, including medical treatment = cost saving with worse scenarios of US $56/LYG and US $262/acute MI averted	Cost saving for complete smoking ban	Indian Rupees, 2008 and converted to US$
Ekwunife et al. 2013	Nigeria	7	Assessed the CE of 4 anti-HTNsive med [Diuretic, BB, ACEi, CCB] for treating hypertensive patients 40 years and above based on 3 CVD risk levels from Framingham equations compared to no treatment	Primary	Individual	Thiazide (RR stroke = 0.63, RR CHD = 0.84, RR death = 0.89); Propranolol (RR stroke = 0.83, RR CHD = 0.90, RR death = 0.96); Lisinopril (RR stroke = 0.65, RR CHD = 0.81, RR death = 0.83); Nifedipine (RR stroke = 0.58, RR CHD = 0.77, RR death = 0.86)	Meta-analysis	Low CVD risk [Thiazide = $2600/QALY], Moderate risk [Thiazide = $1300/QALY], High risk = $Thiazide = $1300/QALY; CCB = $12,500/QALY)	Only Thiazide was CE at all risk levels & CCB at high risk. Rest of drugs were not CE at all risk levels	US dollar 2010
Garcia-Pena et al. 2002	Mexico	3	Assessed the CE of fortnightly nurse home visits to elderly (> 60 years) with HTN (BP ≥ 160/90 mmHg) during 6 months compared to usual care provided by family physicians	Primary	Individual	Not mentioned	–	SBP = 10.46 Pesos (US $1.14)/mmHg drop and DBP = 9.43 Pesos (US $1.03)/mmHg	Highly cost-effective	Mexican pesos, 1998
Gaziano et al. 2015	Mexico, Guatemala, South Africa	3, 7	Assessed the use of paper-based screening tool, mobile app based screening tool for identifying individuals with high CVD risk by community health workers compared to standard care (opportunistic screening)	Secondary	Individual/high risk	Primary prevention: RRR statin [IHD = 0.77, CVA = 0.83], Aspirin [IHD = 0.82, CVA = 0.95] BP treatment [IHD = 0.84, CVA = 0.64]; Secondary Prevention: RRR statin [death = 0.91, MI = 0.69, CVA = 0.81], Aspirin [death = 0.91, MI = 0.69, CVA = 0.81], ACEi [death = 0.87, MI = 0.83, CVA = 0.78], BB [death = 0.94, MI = 0.89, CVA = 0.84]	Meta-analysis of RCTs	Mobile app most CE: $565/QALY in Guatemala, $3.57/QALY in Mexico and cost-saving in South Africa	Cost-effective	US dollar 2013
Gaziano et al. 2005	South Africa	7	Compared CE of various BP guidelines; 2 BP level (the 1995 SA HTN guideline i.e. treat all BP > 160/95 mmHg or 140/90 mmHg with DM, current 2001 guideline of treating BP > 140/90 mmHg or 130/85mmH with DM) and 4 absolute CVD risk strategies against no treatment in adults 35–74 years old	Primary	Individual	Hypertension treatment resulted in 10 mmHg reduction in SBP, 14% (14–25%) risk reduction for IHD & 40% (10–50%) risk reduction for stroke	Meta-analyses of RCTs	10 year absolute CVD risk > 40% ($700/QALY), 30% ($1600/QALY), 20% ($4900/QALY), 15% ($11,000/QALY). Blood pressure level guidelines were dominated (not cost effective)	Absolute risk = cost effective, BP level = not cost-effective	US dollar, 2001
Gaziano et al. 2006	6 World bank regions	All	Compared multidrug treatment for primary CVD prevention in four groups with different thresholds for 10 year absolute risk for CVD and only in one group for secondary prevention	Primary + secondary	Individual	Primary prevention: RR for Aspirin[IHD = 0.68 (0.60–0.77), stroke = 0.84 (0.75–0.93)]; ACEI and CCB[IHD = 0.66 (0.60–0.71), stroke = 0.51 (0.45–0.58)]; Statin = [IHD = 0.64 (0.55–0.74), stroke = 0.94 (0.78–1.14)]//Secondary prevention: RR for Aspirin [IHD = 0.66 (0.6–0.72), stroke = 0.78 (0.72–0.84), BB[IHD = 0.73 (0.75–0.87), stroke = 0.71 (0.68–0.74)], ACEI[IHD = 0.80 (0.70–0.90), Stroke = 0.68 (0.56–0.84)], Statin[IHD = 0.71 (0.62–0.82), Stroke = 0.81 (0.66–1.00)]	Meta-analysis of RCTs	For primary prevention: US $746–890/QALY for patients with 10 year absolute risk of CVD > 25% and $1039–1221/QALY for those with absolute risk > 5%. For secondary prevention: $306/QALY gained	Cost-effective across all 6 world bank regions	US dollar 2001
Gonzalez-Diaz et al. 2015	Mexico	3	Assessed CE of DES (Early generation drug eluting stent [DES] (EGDES) and New generation DES (NGDES) vs. bare metal stent [BMS] in patients with ischemic cardiomyopathy undergoing angioplasty	Secondary	Individual	Risk of major adverse cardiac event: BMS = 01900 (0.1775–0.2144), EGDES = 0.0904 (0.0783–0.1013), NGDES = 0.0764 (0.0410–0.0917)	Meta-analyses of RCTs	EGDES = 28,910/MACE; NGDES = 35,591/MACE; NGDES-EGDES = 84,983/MACE	EGDES and NGDES were cost-effective but not so much for changing from old (EGDES) to new (NGDES) technology	US dollar, 2014
Gu et al. 2015	China	1	Assess CE of treating high BP in people with IHD and stroke (secondary prevention), and two strategies for primary prevention (treat all stage 2 HTN patients and treat all stage 1 and 2 HTN patients) using low-cost anti-hypertensives compared to the status quo	Primary +secondary	Individual	RR per 10 mmHg reduction in SBP or 5 mmHg reduction in DBP: 35–64 years [CHD = 0.73 (0.70–0.77), Stroke = 0.64 (0.59–0.69)], ≥ 65 years [CHD = 0.77 (0.74–0.79), Stroke = 0.69 (0.64–0.74)]; SBP lowering, median effect (change in mmHg) in 35–64 years (target 140 mmHg): Stage 2 HTN(≥ 160 mmHg) = 22.7 (17.5–27.9), Stage 1 HTN (140–159 mmHg) = 6.5 (4.1–8.9); Median effect in age ≥ 65 years(target 150 mmHg): Stage 2 HTN = 17.8 (13.2–22.4), Stage 1 HTN = 2.6 (1.5–3.7); For DBP effect in isolated diastolic HTN(IDH), for age 35–84 years (target 90 mmHg): Stage 2 IDH (normal SBP, ≥ 100 mmHg DBP) = 12.4 (8.7–16.1), Stage 1 IDH (normal SBP, 90–99 mmHg DBP) = 3.5 (2.5–4.6)	Meta-analysis of trials and prospective studies	Secondary prevention = cost saving; Primary prevention (strategy 1 = CE, strategy 2 = borderline CE)	Cost saving for secondary prevention and CE for primary prevention	International dollar for 2015 & CYN 2015
Ha et al. 2011	Vietnam	1	Population: mass media to reduce salt intake, smoking, cholesterol concentration and combined, Individual: education and treatment for high SBP > 140 and > 160 mmHg, cholesterol & combination for absolute CVD risk thresholds	Primary	Population + individual	Mass media for reduce salt intake = − 20% (10–30%); mass media to reduce prevalence of smoking = − 1.5% (0.8–2.3%); mass media to reduce cholesterol = − 2% (1–3%); education and individual treatment of BB and diuretic for SBP > 140 and > 160 mmHg for difference from actual SBP and 115 mmHg = − 28% (23–33%); individual treatment for cholesterol with statins = – 20% (17–23%)	Systematic review of RCTs and prospective cohort studies	Population intervention media campaign for salt reduction = VND 1945002/DALY or US $118/DALY and Individual treatment for SBP > 160 mmHg = VND 1281596 or US $78/DALY averted most cost effective	Cost effective	Vietnamese Dong, 2007
Huang and Ren 2010	China	1	Assessed the cost–benefit of preventing stroke via treatment of hypertension	Secondary	Population	–	–	CB ratio = 1:3.57	Cost effective	Chinese Yuan Renminbi (CNY), 1997
Jafar et al. 2011	Pakistan	6	Compared the CE of 3 intervention groups to reduce BP (home health education alone, GP training alone, HHE and GP training) versus no intervention/usual care	Primary	Individual	5 mmHg reduction in BP assumed to lead to 20% reduction in CVD DALYs	Meta-analysis of RCTs and prospective study	HHE and GP training most cost-effective = $23/mmHg reduction in SBP	Cost effective	Pakistan rupees, converted to US $2007
Jarungsuccess et al. 2014	Thailand	1	Compared the CE of various New oral anticoagulants (NOACs)[Rivaroxaban, Apixaban, Dabigatran] vs. warfarin in preventing stroke in patients 65 years plus with non-valvular AF	Primary	Individual	For Ischaemic stroke: RR of dabigatran 150 mg BID = 0.55 (0.32–0.95), RR dabigatran 110 mg BID = 1.01 (0.63–1.61), RR Rivaroxaban 20 mg OD = 0.82 (0.55–1.22), RR Apixaban 5 mg BID = 0.65 (0.32–0.98); For MI: RR Dabigatran 150 mg BID = 0.89 (0.80–0.98), RR Dabigatran 110 mg BID = 0.90 (0.01–1.80), RR Rivaroxaban 20 mg OD = 0.80 (0.54–1.06), RR Apixaban 5 mg BID = 0.88 (0.60–1.16)	RCTs	Govt perspective (GP), societal perspective (SP):: Dabigatran 150 mg = 2268,738.48/QALY for GP and 2,252,938.19/QALY for SP; Dabigatran 110 mg = 46,426,823.22/QALY for GP and 46,286,254.56/QALY for SP; Rivaroxaban 20 mg = 5,050,231.84/QALY for GP and 5,030,280.45/QALY for SP; Apixaban 5 mg = 5,583,860.99/QALY for GP and 5,565,388.48/QALY for SP	Not cost effective	Thai baht 2013
Khonputsa et al. 2012	Thailand	1	Compared several BP (diuretic, ACEI, CCB, ARB) and lipid(statin) lowering medication singly and in combination as well as theoretical polypill in preventing IHD & stroke via absolute CVD risk approach	Primary	Individual	RR for Diuretic[IHD = 0.86 (0.75–0.98), IS and HS = 0.62 (0.53–0.72)]; ACEI [IHD = 0.83 (0.78–0.89), IS and HS = 0.78 (0.66–0.92)]; B-blocker[IHD = 0.89 (0.78–1.02), IS and HS = 0.83 (0.70–0.99)]; CCB [IHD = 0.78 (0.62–0.99), IS and HS = 0.66 (0.58–0.75)]; ARB [IHD = 0.86 (0.53–1.40), IS and HS = 0.79 (0.69–0.90)]; Statin [IHD = 0.77 (0.74–0.80), IS = 0.78 (0.70–0.87), HS = 1.00]; Polypill[IHD = 0.44 (0.34–0.54), IS = 0.32 (0.24–0.41), HS = 0.41 (0.31–0.52)]	Meta-analysis of RCTs	Polypill was dominant (i.e. − 10,909/DALY) and combination of 3 antiHTNsive (D + CCB + ACEI) was dominant (i.e. − 1573/DALY) in all 10 year CVD risk levels [5–9%, 10–19% and ≥ 20%] evaluated. Adding statin to the mix of anti-HTNsives increased ICER progressively from 45,000 to 130,000 TB/DALY	Triple anti-HTNsive = Cost saving at all CVD risk levels, but CE with addition of statin	Thai baht, 2004
Lakic et al. 2012	Serbia	2	Compared CE of 4 anti-HTNsives used in clinical practice (diuretic, ACE-I, BB, CCB) with no intervention and with each other to identify which was most CE to initiate as monotherapy	Primary	Individual	Not clear	–	Diuretic = €74.27/QALY, BB = 75.58/QALY, ACE-I and CCB were dominated	Diuretic was most cost-effective to initiate as monotherapy	Serbian dinar 2009, converted and presented in Euros
Li et al. 2015	China	1	Assessed the CE of clopidogrel compared with aspirin in patients with ischemic stroke and peripheral artery disease	Secondary	Individual	Relative risk reduction of ischemic stroke, MI or vascular death of 8.7% (95% CI 0.3–16.5)	RCT (CAPRIE trial)	Ischemic stroke = $US 5246/QALY and 0.9LY per patient; PAD = $US 9890/QALY and 0.28LY per patient for clopidogrel compared to aspirin therapy	Cost-effective	US dollar 2013
Mason et al. 2014	Tunisia, Syria, Palestine and Turkey	2, 4	3 salt reduction policies (health promotion, voluntary labelling of food, mandatory reformulation) to reduce CHD mortality	Primary + secondary	Population	Health promotion (HP) = 5% (1–35%), food package labelling = 10% (5–15%), mandatory reformulation = 10% (5–40%), HP + labelling = 15% (10–20%), HP + reformulation = 15% (15–30%), All 3 policies = 30% (10–50%)	Systematic reviews & Meta-analysis	Turkey = all policies cost-saving. Tunisia = all policies cost-saving except HP = $15,377/LYG. Syria = HP and labelling cost saving except reformulation. Combining reformulation and HP + labelling became cost-saving. Palestine = all policies cost-saving except reformulation	Cost saving	local currency converted to Int$, 2010
Mejia et al. 2015	Colombia	3	Compared the CE of Ticagrelor versus clopidogrel for treatment of patients with acute coronary syndrome to prevent future MI and stroke	Secondary	Individual	RR of death after MI (after 1 year = 5.84, long-term = 2.21), RR of death after stroke (year 1 = 7.43, long-term = 2.07)	RCT	COP$ 28,411,503/QALY gained	Cost-effective	Colombian pesso (COP$), 2010
Murray et al. 2003	Multiple	3, 6	Assessed CE of a range of population (voluntary & legislative salt reduction and health education for BMI and cholesterol) and individual (treatment for HBP and cholesterol, absolute CVD risk) in preventing CVD events	Primary	Population + individual	Effectiveness: voluntary salt reduction = 15% reduced intake with BP changes, salt legislation processed foods = 30% reduced intake; Health education for BMI and cholesterol = 2% drop in cholesterol; HTN treatment (160 mmHg and 140 mmHg) with BB + diuretic and education = 33% reduction in difference between actual SBP and 115 mmHg, Statin for high total cholesterol (> 6.2 mmol/L and > 5.7 mmol/L) and education = 20% drop in total cholesterol; Absolute risk = combined effect of BP and cholesterol treatment + 20% reduction of CVD risk for antiplatelet therapy	Meta-analysis of RCTs	Latin America: Legislation salt reduction = Int$13/DALY, Salt legislation to health educ for cholesterol = Int$14/DALY, Combined population and interventions = Int$29 − 432/DALY; South east Asia: Health education for cholesterol = Int$14/DALY, Health educ for cholesterol to Combined salt legislation + health educ = Int$20/DALY; Combined population and individual intervention with absolute risk = Int$24− 206/DALY	Cost-effective	Int Dollar
Ngalesoni et al. 2016	Tanzania	7	Compared the CE of various drugs (Captopril, Losartan, Atenolol, Nifedipine, Bendrofluazide, Aspirin, Simvastatin, Metformin, Glibenclamide) singly or combinations in absolute CVD risk prevention in those with and without diabetes against no treatment	Primary	Individual	ACE-I [RR MI = 0.81 (0.70–0.94), RR stroke = 0.65 (0.52–0.82)], ARB [RR MI = 0.94 (0.85–1.03), RR stroke = 0.91 (0.85–0.98)], BB [RR MI = 0.90 (0.78–1.03), RR stroke = 0.83 (0.72–0.97)], CCB [RR MI = 0.85 (0.78–0.92), RR stroke = 0.66 (0.58–0.75)], Soluble Aspirin [RR MI = 0.77 (0.69–0.86), RR stroke = 0.95 (0.85–1.06)], Statin [RR MI = 0.86 (0.82–0.90), RR stroke = 0.90 (0.85–0.95)], Thiazide diuretic [RR MI = 0.84 (0.75–0.95), RR stroke = 0.63 (0.57–0.71)], Biguanide [RR MI = 0.67 (0.51–0.890, RR stroke = 0.80 (0.50–1.27)], Sulfonylureas [RR MI = 0.85 (0.74–0.97), RR stroke = 0.91 (0.73–1.13)]	Meta-analyses of trials	CVD risk only → VHR [ACEi+CCB+Diu+Sta+ASA] = $652/DALY, [ACEi+CCB+Diu+ASA] = $498/DALY; HR [ACEi+CCB+Diu+Sta] = $607/DALY, [ACEi+CCB+Diu] = $349/DALY; MR [ACEi+Diu+ASA] = $554/DALY, [ACEi + Diu] = $164/DALY; LR [ACEi+Diu+Sta] = $3175/DALY, [ACEi+Diu] = $1327/DALY. CVD risk with Diabetes → VHR [Big+Sulf+ACEi+ARB+CCB+Sta+ASA] = $7615/DALY, [Big+Sulf+ACEi+CCB+Sta+ ASA] = $704/DALY, [Big+Sulf+ACEi+CCB+ASA] = $350/DALY; HR [Big+Sulf+ACEi+ARB+CCB+Sta] = $10300/DALY, [Big+Sulf+ACEi+CCB+Sta] = $914/DALY, [Big+Sulf+ACEi+CCB] = $309/DALY; MR [Big+Sulf+ACEi+CCB+Sta] = $945/DALY, [Big+Sulf + ACEi+CCB] = $256/DALY, [Sulf+ACEi+CCB] = $115/DALY; LR [Big+Sulf+ACEi+CCB+Sta] = $2480/DALY, [Big+Sulf+ACEi+CCB] = $958/DALY, [Sulf+ACEi+CCB] = $608/DALY	For CVD risk without diabetes, medical management was CE at all risk levels except in low risk individuals. For CVD risk with diabetes, combination of Sulfonylurea, ACE inhibitor and Calcium channel blocker in low and moderate risk groups was highly CE. For high risk (adding Biguanide + Statin) and Very high risk (adding Biguanide + Statin + ASA) were similarly CE. Other combinations were not CE	US dollar 2012
Nguyen et al. 2016	Vietnam	1	Assess CE of no HTN screening versus screening in 4 scenarios (one-off, annual (E1), every two years (E2), screening with increased coverage of treatment at different ages	Secondary (screening)	Population	RR of HBP to acute CVD = 0.72, RR of CVD-death = 0.82	Meta-analysis of RCTs	10 year model: Screening at 35 years not CE. One off screening at 45 years was CE (Int$ 12,070/QALY for women and Int$ 4183/QALY for men) and rest of scenarios not CE. Screening for men at 55 years was cost-saving for one-off screen and CE for other scenarios, for women at 55 years = One off = Int$ 871/QALY and Int$7425/QALY in E2 plus 20% treatment cover(TC). || Lifetime model: All scenarios were CE for men all ages; For women = all scenarios were CE except E1 at 35 years, E1 and 20% TC, E2 until 55 years then E1, E2 until 60 then E1	Cost effective for men 55 years and above but varies in women of similar age	International dollar for 2013, converted from VND
Ortegon et al. 2012	2 WHO regions (AfrE and SearD)	6, 7	Assessed 123 single and combined interventions (36 tobacco (individual and population strategies), 77 CVD (population salt reduction strategies and individual HTN and Cholesterol treatment, and treatment based on 10 year absolute CVD risk) compared with do nothing scenario	Primary + secondary	Population + individual	RR for SBP Age 30–44 years (IHD = 1.07, Stroke = 1.09), 45–59 year (IHD = 1.05, Stroke = 1.07), 60–69 (IHD = 1.03, stroke = 1.05), 70–79 year (IHD = 1.02, Stroke = 1.03), ≥ 80 year (IHD = 1.01, stroke = 1.02); RR cholesterol 30–44 years (I = 3.65, S = 1.48), 45–59 years (I = 2.08, S = 1.35), 60–69 (I = 1.55, S = 1.25), 70–79 years (I = 1.42, S = 1.17), ≥ 80 year (I = 1.42, S = 1.09); RR-Smoking: 30–44 and 45–59 years (Stroke = 3.12, IHD and Stroke = 2.43, IHD and COPD = 6.43), 60– ≥ 80 year (Stroke = 1.65, IHD and COPD = 5.73), IHD and Stroke (60–69 years = 1.84, 70–79 year = 1.70, ≥ 80 year = 1.38)	WHO and GBD 1990 study	FCTC demand reduction strategies ≤ $Int950 and < $Int200 per DALY averted in AfrE and SearD respectively; combination therapy for those with > 25% absolute CVD risk ≤ $Int150 and < $Int230 per DALY averted in AfrE and SearD respectively)	Majority were cost-effective	International dollar for 2005
Pan et al. 2014	China	1	Compared the CE of Clopidogrel plus Aspirin in preventing recurrent stroke after TIA versus Aspirin alone	Secondary	Individual	90-day risk of stroke: HR = 0.68 (0.57–0.81), recurrent rate of stroke = 0.1219 (0.1163–0.1276)	RCT & Chinese National Stroke Registry	CNY 33,800 (US $5200)/QALY	Cost-effective	Chinese Yuan Renminbi (CNY), 2011
Permanicha et al. 2015	Thailand	1	Assessed cost-effectiveness (C-E) of n-3 polyunsaturated fatty acids (PUFAs) in addition to standard therapy compared with standard therapy alone in post-MI patients	Secondary	Individual	Risk ratio = 0.73 (0.60–0.89)	Meta-analysis	256,199 Thai baht/LYG and 297,193 Thai baht/QALY. ICER was lower in older (45–85 years) patients	Not cost-effective	Thai baht (THB), inflated to 2013 values using Consumer Price Index (CPI)
Permsuwan et al. 2015	Thailand	1	Assessed the CE of Fondaparinux over Enoxaparin in patients with NSTEMI-ACS	Secondary	Individual	RR of Fondaparinux on major bleeding = 0.52	RCT	Dominant in both societal and provider perspective	Cost saving	Thai baht, 2013
Polanczyk et al. 2007	Brazil	3	Compared the CE of Sirolimus eluting stents (SES) & SES after BMS versus BMS in preventing restenosis events at one year	Secondary	Individual	Restenosis rate for de novo lesion [BMS = 0.30 (0.10–0.50), SES = 0.06 (0.02–0.15) with RR reduction = 80%]	RCT	Private sector: BMS followed by SES = Dominated, SES = R$ 27,403/event avoided; Public sector: BMS followed by SES = Dominated, SES = R$ 47,529/event avoided	Not cost effective	Brazilian reals(R$) in 2003
Rabus et al. 2005	Turkey	2	Assessed CE of TPA versus Streptokinase for thrombolysis in prevention of recurrent CVD event in patients with AMI	Secondary	Individual	–	–	TPA vs SK = €47,289/LY saved	Cost-effective	Euro, 1999
Ribeiro et al. 2010	Brazil	3	Assessed the CE of ICD use in 60 year. old HF patients (NYHA II and III) compared to treatment with standard HF therapy	Secondary	Individual	RR of all-cause mortality from ICD use = 0.74 (0.67 - 0.83)	Meta-analysis of RCTs	US $50,345/QALY and US $44,304/LYS	Not cost effective	Brazilian reals (R$) in 2007 and Int dollars, converted to $US via PPP
Robberstad et al. 2007	Tanzania	7	Compared CE of various drugs (Aspirin, Atenolol, Nifedipine, Lovastatin, HCT) and combinations in 4 absolute risk categories for primary CVD prevention versus do nothing	Primary	Individual	Aspirin [RR stroke = 0.84 (0.75–0.93), RR CHD = 0.68 (0.60–0.77)], Diuretic (HCT) [RR stroke = 0.66 (0.55–0.78), RR CHD = 0.72 (0.61–0.85)], BB (Atenolol) [RR stroke = 0.71 (0.59–0.86), RR CHD = 0.93 (0.80–1.09)], CCB (Nifedipine) [RR stroke = 0.87 (0.77–0.98), RR CHD = 1.12 (1.00–1.26)], Statin (Lovastatin) [RR stroke = 0.83 (0.75–0.91), RR CHD = 0.39 (0.29–0.49)], Hypothetical polypill [RR stroke = 0.20 (0.13–0.29), RR CHD = 0.12 (0.09–0.16)]	RCT and SR of RCTs	Diuretic (HCT) in high risk group = $85/DALY (highly CE), Aspirin+Diuretic = $143/DALY|| Aspirin, BB, CCB, Statin, Aspirin+BB, Diuretic+BB, Aspirin+Diuretic+Statin, Diuretic+BB+Statin, Aspirin+BB+Statin = All were dominated. Hypothetical polypill = $1476/DALY (not CE)	Diuretic alone was highly CE in all risk groups but especially for high risk group, Diuretic + Aspirin was CE in high and medium risk but not low risk group. All other combinations were not CE	US dollar 2005
Rosendaal et al. 2010	Nigeria	7	Assessed the CE of hypertension screening and treatment using 2 strategies (Strategy I: Stage 1 HTN combined with CVD risk < 20% or Stage 2 HTN with any CVD risk level, Strategy II: All HTNsive with 10 year CVD risk > 20%) vs no screening and treatment	Screening	Population	RRR per 10 mmHg SBP decrease(Lawes): 30–44 years [Stroke = 2.38 (2.13–2.63), CHD = 1.92 (1.54–2.38)], 45–59 years [Stroke = 2 (1.92–2.04), CHD = 1.67 (1.56–1.75)], 60–69 years [Stroke = 1.56 (1.52–1.61), CHD = 1.33 (1.27–1.39)], 70–79 years [Stroke = 1.37 (1.32–1.43), CHD = 1.25 (1.19–1.32)]; Rapsomaniki formula: RRR stroke = 1.16 (1.14–1.18), RRR CHD = 1.16 (1.15–1.18)	WHO Global analysis	Strategy I: Framingham = $6282/DALY, Rapsomaniki = $5315/DALY, Lawes = $1287/DALY; Strategy II: Framingham = $2644/DALY, Rapsomaniki = $2221/DALY, Lawes = $634/DALY	Strategy II was more CE compared to Strategy I which was moderate CE and trended to being dominated	US dollar 2012
Rubinstein et al. 2010	Argentina	3	Compared the CE of 2 population (reduce salt in bread and mass media for tobacco cessation) & 4 individual (treatment for HBP, cholesterol, Bupropion for tobacco & Polypill for absolute CVD risk > 20% in 10 years) interventions versus do nothing	primary	Population + individual	Efficacy of interventions == Mass media for tobacco cessation = reduce current smoker prevalence by 7%, RR for reducing salt in bread = 0.99, Bupropion for tobacco cessation = annual cessation rate of 28%, HBP treatment [including atenolol, Enalapril, amlodipine, hydrochlorothiazide] (RR CHD = 0.66, RR stroke = 0.51), Cholesterol lowering treatment [Atorvastatin] = (RR CHD = 0.77, RR stroke = 0.81), Polypill [including Aspirin, Enalapril, Amlodipine, Atorvastatin] for absolute CVD risk > 20% at 10 years = (RR CHD = 0.34, RR stroke = 0.32)	Global and regional analysis, Meta-analyses	Reduce salt in bread = cost saving, Polypill for absolute risk > 20% = cost saving, Treatment for HBP = Int$2977/DALY (was CE), Mass media for tobacco cessation = Int$3186/DALY (was CE), treatment for high cholesterol = Int$14,431/DALY, Bupropion for tobacco = Int$59,433/DALY (not CE)	Salt reduction in bread and absolute risk interventions were cost saving, others were cost effective except Bupropion which was not cost effective	Argentine pesos 2007, coverted to International dollar
Salomon et al. 2012	Mexico	3	Compared CE of range of tobacco (taxation, clean indoor air law, advertising ban, NRT), salt (voluntary industry reduction & legislation to reduce in processed foods), BP (drug treatment and dietary advice), cholesterol (Statin treatment and dietary advice)& absolute CVD risk(Aspirin treatment) interventions against do nothing	Primary + secondary	Population + individual	Tobacco effectiveness: % reduction in consumption [current 60% tax vs. null = − 71.5% (15–30 years and − 57.2% (30+ years old); Increase tax at 80% vs. null = − 79.6% (15–30 years old) and − 63.7% (30+ years old); Clean indoor air laws = − 2.8% (males) and − 0.9% (females); Comprehensive advertising ban = − 5%; Nicotine replacement therapy (NRT) = − 3.1%]; CVD effects[For Salt intake reduction: Voluntary reduction by manufacturers in processed food = − 15%, Legislation to reduce salt in processed food = − 30%; For Cholesterol lowering: Mass media campaign = − 2%, Statin treatment plus education on lifestyle modification with diet advice = − 20%; For BP (difference btw SBP and 115 mmHg): drug treatment plus lifestyle modification with diet advice = − 33%; Absolute CVD risk: aspirin treatment = − 20%]	Systematic review & meta-analysis	For tobacco = Increased taxation was CE Int$103/DALY, rest (NRT, ban, clean indoor law) were dominated. For primary CVD prevention: Population salt reduction by 30% = most CE (Int$210/DALY), Absolute risk, 35% threshold = Int$526/DALY. For secondary CVD prevention: All drug treatment (BB, ACE-I, Statin, Thrombolysis with streptokinase, exercise training) = dominated. Only diuretic (for HF) was CE = Int$590/DALY, Cardiac rehabilitation = Int$38/DALY, All HF interventions = Int$1120/DALY	Tobacco taxation = CE, rest (especially individual NRT) dominated. 30% pop Salt reduction = CE, secondary prevention = dominated except HF interventions & diuretic	International dollar for 2005
Schulman-Marcus et al. 2010	India	6	GP providing pre-hospital ECG for patients with chest pain prior to referral versus no ECG	secondary	individual	GP sensitivity (with ECG = 0.818, no ECG = 0.667), GP specificity (with ECG = 0.5, no ECG = 0.3), RRR thrombolytic = 0.75 | diagnosed MI and CVD mortality	Prospective study & multicentre RCT	$12.65/QALY gained for doing ECG	Cost effective	Indian rupees, 2007 coverted to USdollar 2007
Tolla et al. 2016	Ethiopia	7	Compared the CE of various drugs (Aspirin, ACEi, BB, Streptokinase, ASA + Clopidogrel, PCI) singly or combination for secondary prevention of stroke and MI as well as BP lowering, cholesterol lowering treatment and combination for absolute CVD risk for primary prevention versus do nothing	Primary + secondary	individual	Efficacy of interventions == Primary prevention: anti-HTNsive treatment (SBP > 140 or > 160 mmHg) for difference in SBP and 115 mmHg = 33% (31–44%), Efficacy cholesterol lowering (> 5.7 or > 6.2 mmol/l) for serum level of cholesterol = 20% (17–23%), Combination of treatment for absolute CVD risk (> 5%, > 15%, > 25%, > 35%) for effect on level of SBP = 30%, plus cholesterol = 20% plus Aspirin = 18%; For treatment of acute MI (effect on 28 day mortality): Aspirin = 22% (15–29%), ACEi = 7% (2–11%), BB = 13%(2–23%), Streptokinase = 26% (17–31%), ASA + Clopidogrel = 32% (17–47%), PCI = 61% (38–75%); For post-acute MI (effect on case fatality rate): Aspirin = 13% (2–22%), ACEi = 23% (14–30%), BB = 23% (16–30%), Statin = 19% (15–24%); For acute ischemic stroke (28 day case fatality rate): Aspirin = 5% (1–9%); For post-acute stroke (case fatality rate): Aspirin = 16% (2–29%), ACEi = 16% (12–30%), Statin = 24% (16–37%)	Meta-analysis of RCTs	For primary prevention: Combination treatment for absolute CVD risk > 35% = $67/DALY, absolute risk > 25% = $131/DALY, absolute risk > 15% = $177/DALY, absolute risk > 5% = $341/DALY, rest were dominated. For secondary prevention: post acute stroke − [ASA + Statin + ACEi] = $1061/DALY while rest dominated, post acute IHD = $1849/DALY (not CE) and rest were dominated, Acute MI treatment [ASA + Streptokinase + ACEi + BB] = $999/DALY, rest of treatment combinations were either not CE or dominated	In primary prevention, absolute risk was CE, while BP treatment at 140 or 160 mmHg as well as cholesterol lowering treatment were not CE. Selected combination interventions for secondary prevention were CE while the majority were dominated(not CE)	US dollar 2012
Wang et al. 2013	China	1	Compared CE of optimal use of acute MI treatments within 30 days in the following strategies [A1: use of all 4 oral drugs in patients with AMI, A2: Clopidogrel in AMI, B: Unfractionated Heparin in NSTEMI, C1: PCI in tertiary hospitals & thrombolysis with Streptokinase in secondary hospitals in patients with STEMI, C2: primary PCI in all STEMI patients, C3: primary PCI in high-risk patients with NSTEMI in tertiary hospitals) compared to current practice of non-optimal use in patients with AMI	Secondary	Individual	RR Aspirin 75 mg daily, 30 days = 077 (0.70–0.89), RR BB (Atenolol 50 mg daily) 30 days = 0.88 (0.80–0.98), RR ACE-I (Captopril 50 mg daily) 30 days = 0.94 (0.89–0.98), Statins (Simvastatin 40 mg daily) 30 days = 0.77 (0.59–1.01), Clopidogrel (300 mg loading dose, 75 mg daily till 30 days) = 0.93 (0.87–0.99), IV unfractionated heparin (1200 U hourly, 3 days) for NSTEMI patients = 0.84 (0.36–1.98), Thrombolysis with Streptokinase for STEMI patients = 0.75 (0.71–0.79), PCI for STEMI = 0.50 (0.35–0.71), PCI for NSTEMI = 0.75 (0.63–0.90)	Observational, RCT, Meta-analysis of trials & Cochrane review	Strategy A1 = $3100/QALY, Strategy B = $2800/QALY, Strategies C1 = $9000/QALY, C2 ≤ $10,700/QALY (NB: C1 and C2 were moderately CE, while A1 and B were highly CE); Combination of A1 + B = $3000/QALY, Combination of A1 + B+C1 = $8900/QALY and were highly and moderately CE respectively. Other strategies (A2 and C3) not cost-effective	NB: Strategy C1 & C2 were moderately CE, while A1 and B were highly CE); Other strategies (A2 and C3) not cost-effective	US dollar 2013
Wang et al. 2017	China	1	Assessed the CE of treating adult patients in rural community with Nitrendipine-Hydrochlorothiazide (NH) versus Nitrendipine-Metoprolol(NM) on BP reduction	Primary	Individual	Not mentioned	–	NH = $1.4/mmHg for SBP & $2.8/mmHg; NM = $1.9/mmHg for SBP & $3.8/mmHg	NH was more CE than NM	US dollar 2013
Wilcox et al. 2015	Syria	4	3 salt reduction policies (health promotion, voluntary labelling of food, mandatory reformulation) and combinations compared to no salt reduction policies	Primary	Population	%reduction in daily salt intake: health promotion(HP) = 5% (1–35%), labelling salt content(L) = 10% (5–15%), reformulation salt content (R) = 10% (5–40%), R + HP = 15% (10–20%), R + L = 15% (15–30%), R + HP + L = 30% (10–50%)	Cochrane review, Policy analysis	HP, L, and R + HP + L were cost saving	Cost saving	International dollar for 2010
Wu B et al. 2014	China	1	Assessed the CE of Rivaroxaban vs. warfarin, vs. Aspirin, vs. Aspirin + Clopidogrel, vs. no prevention in adults with AF stratified into 7 CHADS2 scores categories	Primary	Individual	RR for IS [Warf in target vs. no = 0.25 (0.06–0.44), Warf INR < 2 vs. no = 1 (0.8–1.2), Warf INR > 3 vs. no = 0.25 (0.06–0.44), aspirin vs. no = 0.81 (0.65–0.99), Aspirin + Clopidogrel vs. aspirin = 0.72 (0.62–0.83), rivaroxaban vs. warfarin all range = 0.94 (0.75–1.17)]; RR of ICH [no vs. warfarin all range = 0.330 (0.264–0.396), aspirin vs. Warf all range = 0.64 (0.50–0.80), Aspirin + Clopidogrel vs. aspirin = 1.37 (0.79–2.37), rivaroxaban vs. Warf all range = 0.67 (0.47–0.93)]; RR of MI[Warf INR < 2 vs. target range = 3.87 (3.87–3.99), Warf INR > 3 vs. target range = 1 (0.8–8.28), rivaroxaban vs. Warf all range = 0.81 (0.63–1.06)	Cohort studies and Meta-analysis	Rivaroxaban compared with no prevention ($116,884/QALY), vs. Aspirin ($153,944/QALY), vs. Aspirin + Clopidogrel ($155,979/QALY), vs. Warfarin ($216,273/QALY)	Rivaroxaban not cost-effective	US dollar, 2012
Yan et al. 2015	China	1	Compared C-E of rt-PA (recombinant tissue plasminogen activator) used within 6 h of acute ischemic stroke versus usual care according to Chinese treatment guideline for CVD 2007	Secondary	Individual	–	–	¥103,050/utility gained ($14,231/UG) in rt-PA therapy	rt-PA was cost-effective, using threshold of $24,462 [(3xGDP per capita($8154)]	2008 Chinese Yuan (CNY), NB: No inflation done to 2012 (year of study)

Regions: 1 = East Asia and Pacific, 2 = Europe and Central Asia, 3 = Latin America and the Caribbeans, 4 = Middle East and North Africa, 5 = North America, 6 = South Asia, 7 = Sub-Saharan Africa; *CE* cost-effective (ness), *RCT* randomized controlled trial, *CVD* cardiovascular disease, *CVE* cardiovascular event, *OR* odds ratio, *RR* relative risk, *RRR* relative risk reduction, *MI* myocardial infarction, *ACS* acute coronary syndrome, *NSTEMI* non ST segment elated myocardial infarction, *CHD* coronary heart disease, *HTN* hypertension, *PCI* percutaneous coronary intervention, *rt-PA* recombinant tissue plasminogen activator, *HTN* hypertension, *MetS* metabolic syndrome, *DM* diabetes mellitus, *CCB* calcium channel blocker, *BB* beta blocker, *ACEi* ACE inhibitor, *IHD* ischemic heart disease, *BP* blood pressure, *SBP* systolic blood pressure, *SDBP* sitting diastolic blood pressure, *GDP* gross domestic product, *GBD* Global Burden of Disease, *WHO* world health organization

### Quality appraisal

Half of the included studies were of high quality. Of the remaining studies, 21 (42%) classified as moderate quality and 4 (8%) as low quality. Details of the quality assessment can be found on Table 2 and Additional file 4.

**Table 2:** Quality assessment of studies with Drummond’s checklist and UK National Institute for Health and Clinical Excellence (NICE) quality criteria

Author, publication year	Drummond score	NICE quality rating	Risk of bias
Permanicha et al. 2015	24/35	+	Moderate
Anderson et al. 2000	15/35	−	High
Mason et al. 2014	29/35	++	Low
Donaldson et al. 2011	25/35	+	Moderate
Yan et al. 2015	17/35	−	High
Bautista et al. 2013	24/35	+	Moderate
Anderson et al. 2000	21/35	+	Moderate
Basu et al. 2016	29/35	++	Low
Khonputsa et al. 2012	28/35	++	Low
Rabus et al. 2005	22/35	+	Moderate
Gaziano et al. 2006	27/35	++	Low
Gaziano et al. 2015	26/35	+	Moderate
Li et al. 2015	31/35	++	Low
Ortegon et al. 2012	29/35	++	Low
Permsuwan et al. 2015	28/35	++	Low
Ha et al. 2011	30/35	++	Low
Schulman-Marcus et al. 2010	28/35	++	Low
Jafar et al. 2011	29/35	++	Low
Choosakulchart et al. 2013	28/35	++	Low
Lakic et al. 2012	20/35	+	Moderate
Pan et al. 2014	29/35	++	Low
Wilcox et al. 2015	26/35	+	Moderate
Gaziano et al. 2005	27/35	++	Low
Amirsadri and Hassani 2015	31/35	++	Low
Wu et al. 2014	28/35	++	Low
Mejia et al. 2015	25/35	+	Moderate
Salomon et al. 2012	27/35	++	Low
Gu et al. 2015	29/35	++	Low
Nguyen et al. 2016	29/35	++	Low
Davies et al. 2013	24/35	+	Moderate
Jarungsuccess et al. 2014	23/35	+	Moderate
Wang et al. 2013	23/35	+	Moderate
Robberstad et al. 2007	26/35	+	Moderate
Ngalesoni FN et al. 2016	28/35	++	Low
Tolla et al. 2016	27/35	++	Low
Rubinstein et al. 2010	27/35	++	Low
Basu et al. 2015	26/35	+	Moderate
Rosendaal et al. 2010	28/35	++	Low
Ekwunife et al. 2013	27/35	++	Low
Amirsadri and Sedighi 2017	29/35	++	Low
Wang et al. 2017	15/35	−	High
Polanczyk et al. 2007	22/35	+	Moderate
Garcia-Pena et al. 2002	21/35	+	Moderate
Ribeiro et al. 2010	28/35	++	Low
Araujo et al. 2008	21/35	+	Moderate
Araujo et al. 2007	24/35	+	Moderate
Murray et al. 2003	25/35	+	Moderate
Akkazieva et al. 2009	21/35	+	Moderate
Gonzalez-Diaz et al. 2015	26/35	+	Moderate
Huang and Ren 2010	13/35	−	High

Drummond summary score: ≥ 27/35 (75%) = ‘++’, 18–26/35 (50–75%) = ‘+’, score < 18/35 (< 50%) = ‘−’

### Evidence on interventions and their cost-effectiveness

#### Primary prevention

All but four studies evaluating legislative or health education interventions [24, 25, 33, 34], focused on pharmacological interventions. Most of them targeted individuals, with just two exclusive population-based [24, 25] and three targeting both individuals and populations [29, 31, 32].

##### Blood pressure lowering interventions

Among studies that evaluated the cost-effectiveness of single anti-hypertensive drugs, diuretics were found to be the most cost-effective; for initiation as monotherapy [35], for use in high risk groups [16], and at various absolute CVD risk levels [36]. Other BP-lowering medication had comparatively higher cost-effectiveness ratios or were cost-ineffective [16, 18, 35, 36] except for Candesartan, which was found to be cost-effective compared to other Angiotensin II receptor blockers in South Africa [37].

In studies evaluating combination therapies, most were generally dominant or cost-effective in all tested [38] or some [18, 28, 31, 39, 40] absolute CVD risk thresholds, and in people with SBP > 160 mmHg [29]. In people with diabetes, apart from ACE inhibitors and CCB combinations in low and moderate CVD risk individuals, other BP lowering drug combinations were not cost-effective in Tanzania [17]. Three studies assessed cost-effectiveness of various BP treatment guidelines/strategies. In one, treatment based on the 10-year absolute CVD risk was cost effective, whereas treatments based on SBP levels of > 140 or > 160 mmHg were not cost-effective [41]. A modelled evaluation compared three BP treatment strategies; treatment to target (TTT), benefit-based tailored treatment (BTT) and a hybrid strategy proposed by the WHO. The authors found that BTT was more cost-effective than TTT or the hybrid strategy [42]. Gu et al. found that treatment of individuals with stage 2 hypertension only or those with either stage 1 or stage 2 using low cost anti-hypertensives were cost-effective [43]. In a RCT comparing the impact of home health education alone, GP training alone, or the combination of both versus usual care in reducing SBP, the combination strategy was most cost-effective [33].

##### Cholesterol lowering interventions

Individual drug treatment with statins was found to be dominant at both LDL cholesterol thresholds of 160 and 190 mg/dL [44], highly cost-effective in Iranian men older than 44 years [45] and cost-effective at various CVD risk thresholds [17, 28, 31, 32]. In Vietnam, individual statin treatment for cholesterol levels > 5.7 mmol/L and > 6.2 mmol/L was cost-effective, though less attractive compared to other measures explored [29]. At same cholesterol levels, statin treatment was not cost-effective in Kyrgyzstan [26]. When statin was added to a combination of BP lowering medications, it was found to considerably increase ICERs in Thailand [38]. In one study in Tanzania, individual statin treatment alone or in combination with BP-lowering medication and aspirin in all absolute CVD risk thresholds was not cost effective [16]. At population level, mass media and health education interventions for reducing cholesterol were found to be cost-effective [29, 32].

##### Polypill interventions

Three studies evaluated treatment with the polypill in Latin American countries [46], Thailand [38] and Argentina [31]. In the study among Latin Americans, the polypill consisted of a combination of three anti-hypertensives (thiazide 12.5 mg, atenolol 50 mg, ramipril 5 mg), statin (simvastatin 20 mg) and aspirin 100 mg administered once daily to high risk individuals compared to no polypill. It was found to be cost-effective in high risk women and for men aged ≥ 55 years [46]. In Argentina, the polypill strategy comprised administering a combination of enalapril 10 mg, hydrochlorothiazide 25 mg, atorvastatin 10 mg and aspirin 100 mg to people at various absolute CVD risk levels. This was cost-effective in those with a 10 year CVD risk of ≥ 20% [31]. Finally, in the Thai study, a theoretical polypill intervention was used which consisted of a statin in full dose and three anti-hypertensives (diuretic, calcium channel blocker and ACE inhibitor) in half standard doses versus a do nothing scenario. This intervention was cost-saving in all 10 year CVD risk threshold levels, surpassing combination with 3 individual anti-hypertensive drugs [38].

##### Smoking control interventions

As regards smoking control interventions, most studies explored population-based strategies, including mass media campaigns [26, 28, 29, 31], legislation for smoking bans [24, 28] and increased taxation [28, 30]. Implementing a complete smoking ban compared to a partial smoking ban was cost-saving in India [24], while all mass media campaigns against smoking and increased taxation for tobacco products were cost-effective [28–31]. However, in Mexico, smoking ban and clean indoor air laws were found not to be cost-effective [30]. In the three studies that evaluated individual-level tobacco interventions, treatments with Bupropion [30] and nicotine replacement therapy [28, 31] were found not to be cost-effective.

##### Salt intake reduction interventions

All interventions to reduce salt intake were population-based, and examined health education via mass media campaigns [23, 25, 26, 28, 29], reduction of sodium content in bread [31], or voluntary industry labelling of foods and mandatory reformulation [23, 25, 28, 30, 32]. All health education strategies were found to be cost-effective. The reduction of sodium content in bread was cost-saving; product reformulation and voluntary reduction were similarly cost-effective or cost-saving, especially when implemented in combination.

##### Atrial fibrillation

Two studies assessed the use of oral anti-coagulants in adults with atrial fibrillation (AF) for primary prevention of stroke. In Thailand [47], three new oral anticoagulants (rivaroxaban, apixaban, and dabigatran) were compared with warfarin in adults aged 65 years and above with non-valvular AF while in China [48], rivaroxaban was compared with warfarin, aspirin, aspirin with clopidogrel and no prevention in adults with AF stratified into seven CHADS2 score categories. In both studies, the new oral anticoagulants were not cost-effective.

#### Secondary prevention

Interventions here were predominantly pharmacological, covering single or combination therapies for blood pressure and cholesterol, anti-platelet aggregates, anti-coagulants and thrombolytic therapy in patients with CVD events (myocardial infarction (MI), stroke, heart failure). One study investigated the cost-effectiveness of influenza vaccination in those with ischaemic heart disease. The rest of the studies focused on medical procedures (stents, implantable cardioverter defibrillators (ICD), percutaneous coronary interventions (PCI)).

##### Blood pressure, cholesterol lowering and antiplatelet aggregate interventions

Among studies that evaluated treatment with blood pressure lowering medication only, ACE inhibitors [49] and diuretics [30] were found to be cost-effective or cost-saving [43]. However, other single treatment interventions with beta-blockers and statins were not cost-effective [18, 28, 30]. One study assessed the addition of n-3 polyunsaturated fatty acids to standard therapy in post MI patients for secondary CVD prevention and mortality, and it was not cost-effective [50]. Combination therapies with a range of BP lowering drugs, statin and aspirin were found to be cost-effective in preventing recurrent stroke events, MI or both [18, 40, 51]. However, Tolla and colleagues found that in Tanzania, some selected combinations of BP and cholesterol lowering drugs with aspirin were not cost-effective [18].

Five studies specifically evaluated the cost-effectiveness of antiplatelet drugs. Two of them showed that clopidogrel alone [52] and clopidogrel with aspirin [53] were more cost-effective than aspirin alone. In one study, clopidogrel for secondary prevention of stroke was cost-ineffective [51]. Ticagrelor was also more cost-effective than clopidogrel in patients with acute coronary syndromes in preventing future stroke or MI [54]. In acute coronary syndrome patients undergoing percutaneous interventions, prasugrel was cost-effective in reducing risk of mortality, stroke and MI [55].

##### Anticoagulant and thrombolysis interventions

Seven studies evaluated interventions with anticoagulant or thrombolytic therapies. Tissue plasminogen activator was found to be cost-effective, when used within 6 h of ischemic stroke [56] and when compared to Streptokinase [57]. In one study, prehospital thrombolysis was found to be cost-effective compared to in-hospital use [58]. Streptokinase was moderately cost-effective when used in combination with other BP medication [18, 51], but not cost-effective when used alone [26]. In one study, fondaparinux was found to be cost-saving compared to enoxaparin in patients with non-ST segment elevated MI (NSTEMI) acute coronary syndrome [59].

##### Medical procedures

Four studies evaluated procedures including, PCI [51], stents [60, 61] and ICD [62]. For stents, drug-eluting early generation and new generation stents were cost-effective compared to bare metal stents [61]. In Brazil, though stents were not cost-effective in preventing CVD events, a sensitivity analysis showed favourable ICERs in patients with diabetes and for small vessels needing revascularization [60]. Compared to standard heart failure (HF) therapy, ICD use in those aged 60 years with HF was not cost-effective in Brazil [62]. In China, PCI was not cost-effective in high-risk patients with NSTEMI acute MI [51].

#### Screening interventions

Three studies evaluated hypertension-screening strategies for population-based interventions [20, 22] and individual/high risk individuals [19]. In Nigeria, two strategies were compared to no screening; strategy 1 entailed hypertension screening and treatment for those with stage 1 hypertension (SBP = 140–159 mmHg and/or DBP = 90–99 mmHg) combined with 10-year CVD risk < 20% or stage 2 hypertension (SBP ≥ 160 mmHg and/or DBP ≥ 100 mmHg) with any CVD risk level. Strategy 2 entailed screening and treatment of all hypertensive people with CVD risk > 20%. The second strategy was found to be cost-effective while strategy 1 was only moderately cost-effective with a tendency to be dominated [22]. In Vietnam, four screening scenarios (one-off screening, annual screening, screening every 2 years and screening in combination with increased treatment coverage) were modelled. All scenarios were cost-effective for men. However, for women two-yearly screening and screening at 35 years were not cost-effective [20]. Gaziano et al. evaluated paper-based and mobile app based CVD screening by community health workers compared to standard care (opportunistic screening). The mobile app was cost effective in Mexico and Guatemala and cost-saving in South Africa [19]. One study in India evaluated the cost-effectiveness of general practitioners doing pre-hospital electrocardiograph (ECG) in patients with chest pain for diagnosis of acute coronary syndrome prior to referral, compared to no ECG. They found that this was a very cost-effective strategy estimated at US$13 per QALY gained [21].

### Methods used in economic evaluation

Table 3 summarizes the methods used in the included studies. Overall, cost-utility analysis was most frequently used (n = 29, 58%), followed by cost-effectiveness analysis (n = 14, 28%). Six studies used both CUA and CEA [37, 45, 50, 52, 62, 63]. There was only one cost–benefit analysis [27]. Overall, among the 20 studies which did CEA, life years gained/saved was the predominant benefit measure [23–25, 41, 44, 45, 49, 50, 52, 57, 58, 62, 63], while the rest of the studies either used drop in blood pressure [33, 34, 37, 39], avoided CVD [24, 44, 61] or restenosis [60] event as benefit measure. Out of 38 studies that mentioned their approach to defining an intervention as cost-effective or not, 7 employed the willingness to pay threshold, while the majority (n = 31) used the WHO’s Commission on Macroeconomics and Health (CMH) threshold using the respective countries’ GDP per capita.

**Table 3:** Economic evaluation methods of included studies

Author, pub year	Type of evaluation	Design	Type of modelling/design	Time horizon	Perspective	Discounting (%)	Uncertainty analysis [# iterations]	Currency and year	Method of CE
Permanicha et al. 2015	CUA and CEA	Modelling	Macro (Markov)	Lifetime	Provider	3	Deterministic (one-way) and PSA [1000]	Thai baht, 2013	WTP
Anderson et al. 2000	CEA	Modelling	Pharmacoeconomic analysis	1 year	Private sector healthcare funder	–	Not stated	Rands, 1999	Not stated
Mason et al. 2014	CEA	Modelling	Macro (Markov)	10 years	Healthcare provider	3	Not stated	Int. dollar, 2010	Not stated
Donaldson et al. 2011	CEA	Modelling	Macro	10 years	Societal (no productivity loss estimated)	3	Not stated	US dollar, 2008	Not stated
Yan et al. 2015	CEA	Empirical	Retrospective	Not stated	Healthcare provider	–	One-way sensitivity [-]	Chinese Yuan, 2008	WHO 3xGDP
Bautista LE et al. 2013	CUA	Modelling	Macro (Markov)	Lifetime	Healthcare system	3	One-way sensitivity [–]	US dollar	Not stated
Anderson et al. 2000	CUA and CEA	Modelling	Pharmacoeconomic analysis	3.8 years	Private sector healthcare provider	5	Not stated	Rands, 1999	Not stated
Basu S et al. 2016	CUA	Modelling	Microsimulation	Lifetime	Healthcare provider	3	PSA [10,000]	US dollar, 2005	WHO 3xGDP
Khonputsa et al. 2012	CUA	Modelling	Macro (Markov)	Lifetime	Healthcare	3	PSA [2000]	Thai baht, 2004	WHO 3xGDP
Rabus et al. 2005	CEA	Empirical	Retrospective	1 year	Government	–	PSA [1000]	Euro, 1999	Not stated
Gaziano et al. 2006	CUA	Modelling	Macro (Markov)	Lifetime	Societal (no productivity loss estimated)	3	PSA [Not stated]	US dollar, 2001	WHO 3xGDP
Gaziano et al. 2015	CUA	Modelling	Microsimulation	Lifetime	Healthcare	3	Deterministic sensitivity [–]	US dollar, 2013	WHO 3xGDP
Li et al. 2015	CUA and CEA	Modelling	Micro (discrete-event simulation)	Lifetime	Healthcare	3	Deterministic (one-way) and PSA [1000]	US dollar, 2013	WHO 3xGDP
Ortegon et al. 2012	CUA	Modelling	Macro (Markov)	Lifetime	Healthcare	3	Deterministic (one-way) and PSA [Not stated]	Int. dollar, 2005	WHO 3xGDP
Permsuwan et al. 2015	CUA	Modelling	Macro (Decision tree & Markov)	Lifetime	Societal	3	Deterministic (one-way) and PSA [1000]	Thai baht, 2013	WTP
Ha et al. 2011	CUA	Modelling	Macro (Markov)	Lifetime	Societal	3	Deterministic (one-way) and PSA [1000]	Vietnamese Dong, 2007	WHO 3xGDP
Schulman-Marcus et al. 2010	CUA	Modelling	Macrosimulation	Lifetime	Societal (no productivity loss & transport estimated)	3	Deterministic (one-way) and PSA [Not stated]	US dollar, 2007	WHO 3xGDP
Jafar et al. 2011	CEA	Empirical	RCT	Not stated	Societal	5	Bayesian PSA [1000]	US dollar 2007	WHO 3xGDP
Choosakulchart et al. 2013	CUA	Modelling	Macro (Markov)	Lifetime	Societal	3	Deterministic (one-way) and PSA [10,000]	Thai baht, 2010	WTP
Lakic et al. 2012	CUA	Modelling	Macro (Markov)	Lifetime	Third party payer	5	PSA [10,000]	Serbian dinar, 2009	WHO 3xGDP
Pan et al. 2014	CUA	Modelling	Macro (Markov)	Lifetime	Healthcare	3	Deterministic (one-way) and PSA [10,000]	Chinese Yuan, 2011	WHO 3xGDP
Wilcox et al. 2015	CEA	Modelling	Macro (Markov)	10 years	Healthcare	3	Multiway sensitivity analysis	Int. dollar, 2010	WHO 3xGDP
Gaziano et al. 2005	CEA	Modelling	Macro (Markov)	10 years	Healthcare	3	Deterministic (one-way) and PSA [1000]	US dollar, 2001	WHO 3xGDP
Amirsadri and Hassani 2015	CUA and CEA	Modelling	Macro (semi-Markov)	Lifetime	Healthcare	3	Deterministic (one-way) and PSA [10,000]	US dollar, 2014	WHO 3xGDP
Wu et al. 2014	CUA	Modelling	Microsimulation	Lifetime	Health system	3	Deterministic (one-way) & PSA [1000]	US dollar, 2012	WHO 3xGDP
Mejia et al. 2015	CUA	Modelling	Macro (Markov)	10 years	Healthcare	3	Deterministic (one-way) and PSA [Not stated]	Colombian Peso, 2010	WHO 3xGDP
Salomon et al. 2012	CUA	Modelling	Macro (Markov)	Lifetime	Societal (no productivity loss estimated)	3	Not stated	Int. dollar, 2005	WHO 3xGDP
Gu et al. 2015	CUA	Modelling	Macro (Markov)	10 years	Healthcare	3	Deterministic (one-way) and PSA [1000]	Int. dollar, 2015	WHO 3xGDP
Nguyen et al. 2016	CUA	Modelling	Macro (Decision tree and Markov)	10 years and Lifetime	Health service	3	Deterministic (one-way) and PSA [5000]	Int. dollar, 2013	WTP
Davies et al. 2013	CUA	Modelling	Macro (Markov)	Lifetime	Healthcare system	3	Deterministic sensitivity analysis [–]	Euros, 2011	WTP
Jarungsuccess et al. 2014	CUA	Modelling	Macro (Markov)	Lifetime	Government and Societal (no productivity loss estimated)	3	PSA [5000]	Thai baht, 2013	WHO 3xGDP
Wang et al. 2013	CUA	Modelling	Macro (Markov)	Lifetime	Societal (no productivity loss estimated)	–	Deterministic (one-way) and PSA [1000]	US dollar, 2013	WHO 3xGDP
Robberstad et al. 2007	CUA	Modelling	Macro (Markov)	Lifetime	Healthcare	3	Deterministic (one-way) and PSA [5000]	US dollar, 2005	WHO 3xGDP
Ngalesoni et al. 2016	CUA	Modelling	Macro (Markov)	Lifetime	Provider and Societal (no productivity loss estimated)	3	Deterministic (one-way) and PSA [Not stated]	US dollar, 2012	WHO 3xGDP
Tolla et al. 2016	CUA	Modelling	Macro (Markov)	Lifetime	Healthcare provider	3	Deterministic (one-way) and PSA [1000]	US dollar, 2012	WHO 3xGDP
Rubinstein et al. 2010	CUA	Modelling	Macro (Markov)	5 years	Healthcare system	3	PSA [1000]	Int. dollar, 2007	WHO 3xGDP
Basu et al. 2015	CUA	Modelling	Micro (Discrete-event simulation)	20 years	Societal (no productivity loss estimated)	3	PSA [10,000]	US dollar, 2014	WHO 3xGDP
Rosendaal et al. 2010	CUA	Modelling	Macro (Markov)	10 years	Healthcare provider	3	Deterministic (one-way) and PSA [1000]	US dollar, 2012	WHO 3xGDP
Ekwunife et al. 2013	CUA	Modelling	Macro (Markov)	30 years	Third party payer	3	PSA [1000]	US dollar, 2010	WTP
Amirsadri and Sedighi 2017	CUA and CEA	Modelling	Macro (Markov)	Lifetime	Healthcare provider	3	Deterministic (one-way) and PSA [Not stated]	US dollar, 2015	WHO 3xGDP
Wang et al. 2017	CEA	Empirical	RCT	Not stated	Healthcare	–	Not stated	US dollar, 2013	Not stated
Polanczyk et al. 2007	CEA	Modelling	Macro (Decision tree and Markov)	1 year and lifetime	Private and public health payers	3	PSA [10,000]	Brazilian reais, 2003	Not stated
Garcia-Pena et al. 2002	CEA	Empirical	RCT	Not stated	Health service and patient	–	Not stated	Mexican pesos,1998	Not stated
Ribeiro et al. 2010	CUA and CEA	Modelling	Macro (Markov)	20 years	Public healthcare system	3	Deterministic (one-way) and PSA [1000]	US dollar, 2007	WHO 3xGDP
Araujo et al. 2008	CEA	Modelling	Macro (Markov)	1 and 20 years	Healthcare system	–	Not stated	Brazilian reais, 2005	Not stated
Araujo et al. 2007	CEA	Modelling	Macro (Markov)	20 years	Healthcare system	7	Not stated	Brazilian reais, 2007	Not stated
Murray et al. 2003	CUA	Modelling	Macro (Markov)	Lifetime	Not stated	3	Multivariate sensitivity analysis [–]	Int. dollar,	WHO 3xGDP
Akkazieva et al. 2009	CUA	Modelling	Macro (Markov)	10 years	Not stated	–	Not stated	Kyrgygstan Som, 2005	WHO 3xGDP
Gonzalez-Diaz et al. 2015	CEA	Empirical	retrospective	Not stated	Health service provider	–	Deterministic (one-way) and PSA [1000]	US dollar, 2014	WTP
Huang and Ren 2010	CBA	Empirical	Retrospective	Not stated	Healthcare	–	Not stated	Chinese Yuan, 1997	Not stated

*CUA* cost-utility analysis, *CEA* cost-effectiveness analysis, *CBA* cost–benefit analysis, *RCT* randomized controlled trial, *PSA* probabilistic sensitivity analysis, *WTP* willingness to pay, *WHO* World Health Organization, *GDP* Gross domestic product

Overall, 43 studies were modelled economic evaluations, while seven were empirical studies with three economic evaluations conducted with randomized trials [33, 34, 39] and four alongside observational studies [27, 56, 57, 61]. For the modelling studies, the majority used a macrosimulation approach, mostly Markov models, with three incorporating decision trees [20, 59, 60]. Among the five studies that used microsimulations, two specifically used discrete-event simulation [52, 64] while the others [19, 42, 48] did not state the technique used.

With respect to study time horizon, 27 studies evaluated interventions over the lifetime of the study population. Six studies did not state the time horizon [27, 33, 34, 39, 56, 61] while the remaining studies (n = 17) varied from one to 30 years.

The majority of studies used a healthcare perspective. A societal perspective was used in 12 studies; however in eight, there was no estimation of productivity loss [17, 21, 24, 30, 40, 47, 51, 64]. Two studies used the third party payer perspective [35, 36], one used the patient perspective [34] and two did not state their perspective [26, 32].

As regards discounting, 40 out of the 50 used discounting for cost and outcomes, most (n = 37) used 3% as their discounting factor. Two of them used 5% [33, 35] and one used 7% [44].

Uncertainty analysis was performed in 40 studies, with the majority doing deterministic (one-way) and probabilistic sensitivity analysis. Ten studies did not state or incorporate any uncertainty around their ICER estimates [23, 24, 26, 27, 30, 37, 39, 44, 49, 58].

In all, 34 studies received some form of funding, including four cases that were funded by pharmaceutical companies [37, 52, 59, 60]. Seven studies did not receive funding while nine did not mention any funding details.

## Discussion

The evidence on cost-effectiveness of interventions for CVD prevention is growing rapidly, with the majority of studies being modelled economic evaluations in the middle-income countries. Primary prevention studies outnumbered those for secondary prevention. Most economic evaluations were for pharmacological interventions focusing on blood pressure, cholesterol lowering and antiplatelet aggregants. BP lowering interventions (mostly diuretics and its combinations) were cost-effective, especially in high risk populations. While some cholesterol lowering interventions alone were not cost-effective, treatment interventions based on absolute CVD risk were mostly cost-effective, with the polypill being most economically attractive. Population-based interventions were few and mostly targeted reduction in sodium intake and tobacco control strategies, and were usually cost-saving.

We observed that the number of publications on economic evaluations for CVD prevention have steadily increased, especially during the last decade. This coincides with, and might arguably be thanks to, the efforts of the Disease Control Priorities Project (DCP2) in 2006, which explored among others the cost-effectiveness of various interventions to combat NCDs. Additionally, the earlier publication of the WHO guide to cost-effectiveness analysis in 2003 [6], and availability of WHO-CHOICE methods [65] are likely catalysts for this observed surge in publications.

For primary prevention, the majority are pharmacological interventions and target high blood pressure, high cholesterol and antiplatelet therapy either singly or in combination. Individual strategies focusing on BP lowering therapies have shown that compared to other antihypertensive drug classes, diuretics are consistently the most cost-effective as monotherapy. Other classes like beta-blockers, ACE inhibitors and calcium channel blockers tend to be favourable mostly when used in combination. Individual treatments with statins are cost-effective in some settings and are not in others, in part due to the different statin drugs evaluated with differing prices across countries. Studies that have evaluated the hypothetical polypill show that it is a very cost-effective option. However, controversy still looms as regards large scale implementation especially in relation to consequences/side-effects of mass treatments and stretching of limited budgets in LMICs [7].

Secondary prevention strategies are similarly geared towards pharmacological strategies, and besides blood pressure and cholesterol lowering interventions; there has also been some focus on thrombolysis and medical procedures. Pharmacological interventions are mostly cost-effective, though with some specifics worth considering. Population-based interventions are relatively few but are cost-effective and or cost-saving. Differences in demographics and epidemiology, modelling assumptions, intervention costs and effectiveness across settings, economic perspectives and time horizons for which interventions are assessed and variation in compliance levels, likely account for the dissimilar conclusions across studies.

Other individual strategies to control smoking like treatments with Bupropion and nicotine replacement therapy are not cost-effective options in the LMICs, although some reports from HICs have shown promise [66].

Population-based interventions have mostly focused on reduction in salt (sodium) intake and smoking. These appear to be the most attractive population-wide interventions, being either very cost-effective or cost-saving in CVD prevention. In a recent systematic review, Hope and colleagues [67] summarized the evidence on economic evaluations of population-based sodium reduction interventions. Similar to our findings, they highlighted that salt reduction interventions offer good value for money. However, similar to ours, they noted that there are few studies assessing the impact of salt tax legislation [67]. Most of the salt reduction interventions focused on health education via mass media campaigns, product reformulation and relabeling.

With respect to tobacco smoking control strategies, contrary to a previous review [66] that suggested majority of interventions focused on nicotine replacement therapy (NRT) and self-help therapies, we found that mass media campaigns, increasing taxes and smoke-free laws were the predominant interventions studied. It is likely that the search strategy and comparatively limited number of databases searched in the prior review, coupled with a focus on high-income countries, might explain the difference. It should be noted, however that we found no economic evaluations of school-based cessation programs, smoking quitlines and tobacco control programs in pregnant women, which have been shown to be cost-effective and potentially cost-saving elsewhere [68–70]. The absence of such economic evidence might be due to the non-existence of such programs or studies evaluating them in LMICs. This constitutes a gap in the strategies to tackle the tobacco epidemic.

With respect to medical procedures, we found very few studies have assessed their cost-effectiveness in LMICs, with the available studies mostly done in Latin American countries. In Brazil for example, early and new generation stents were considered cost-effective, though with limited benefit for moving from early to the new generation stents. Considering the limited available evidence here and the fact that many other regions have not evaluated the use of stents and ICDs, it is difficult to draw reasonable conclusions. However, on a case by case basis, clinicians will be required to strike a balance between long term clinical efficacy and costs to patients and health system.

Screening strategies have been less well explored compared to other interventions. The few existing studies suggest that some strategies are potentially cost-effective. In a bid to enhance their economic attractiveness, their implementation must be stratified for specific population age groups and gender, as well as tailored to account for countries’ specific needs.

As observed in previous reviews [7, 9], there are still few economic evaluations of interventions targeting other risk factors like physical activity, alcohol consumption and body mass. These are established drivers for CVD, and it is important that future studies should consider evaluating interventions targeting those drivers, so as to provide broad perspectives for consideration in stemming the CVD burden.

The majority of included studies are modelled evaluations, with the majority using Markov modelling. This modelling approach has been widely discussed to be suited in modelling chronic diseases such as cardiovascular disease [71]. While model-based evaluations might not be same as real life situations, they are increasingly gaining place in economic evaluation, for a number of reasons. Firstly, economic evaluations conducted alongside RCTs are likely to be limited in time horizon as it is costly for trials to extend for several years [72]. Secondly, the majority of RCTs have intermediate endpoints (such as change in BP or change in cholesterol) as their outcome and very few extend to final end points (CVD event or death, let alone QALYs or DALYs). As such, these are unlikely to reveal the complete picture of costs and benefits of an intervention. Model-based evaluations have the potential to address these problems by using long time horizons [72]. This is particularly seen for smoking-related interventions whose benefits generally accrue in the fourth or fifth decades following implementation of the intervention [73]. Contrary to previous reviews, which found no cost–benefit analysis, we found a single study using this evaluation method. While there is clearly a dearth in studies using this method for evaluation, cost–benefit analyses are likely to be also relevant to policy makers as it allows for direct comparison of health interventions with interventions in other sectors [13].

Up to one-fifth of included studies either did not assess, or failed to incorporate, uncertainty around their ICER estimates. This is particular, as most of the parameters used in modelling studies come from multiple sources, from contexts that differ from those of the target population. It is important to determine the uncertainty around the benefits and costs, and how this affects the ICER estimates. The uncertainty around the cost-effectiveness of interventions is important for policy makers, as they broadly assess and compare the potential gains or losses from implementing one intervention over another [74].

About two-thirds of included studies received some form of funding, mostly from government ministries and universities or educational institutes. We noted that four studies were funded by pharmaceutical industries. Lundh and colleagues in a Cochrane review discussed the impacts of industry funding on research outcomes, in which they highlight that most industry-funded trials are likely to report drugs as efficacious or less harmful [75]. This bias is similarly likely to occur in economic evaluation studies, with such [industry-funded] studies likely to report an intervention or drug as being cost-effective. It is difficult to say with certainty the accuracy of conclusions drawn from the four studies in our review which received pharmaceutical industry funding; with two having low risk of bias [52, 59] and two of moderate risk [37, 60]. It is possible that eliminating these studies, especially those with moderate risk may potentially influence some of our conclusions. We again highlight that interpretation of such findings should be done with caution.

As regards methods for defining an intervention as cost-effective or not, the majority of studies used the WHO Commission on Macroeconomics and Health approach of multiples of GDP per capita, and only very few used a priori willingness-to-pay thresholds. While the proposed WHO method is good at determining those interventions that have good or very good value for money, Bertram and colleagues recently argued about the misuse of these thresholds for decision-making [76]. Modelled cost-effectiveness ratios are amongst others dependent on the construct and validity of the models, variable sources of input parameters; they suggest that for priority setting, decision makers should, besides cost-effectiveness thresholds, take into account other factors such as budget impact, affordability, feasibility of implementation and fairness [76]. Similarly, Remme and co-workers have recently proposed a multi-sectoral perspective for resource allocation, arguing that multiple sectors potentially contribute to health gain and that the goods and services obtained from health sector or interventions can have multiple benefits outside health [77].

In a number of LMICs, Health Technology Assessment (HTA) is currently being considered to guide policy makers in priority setting for the allocation of scarce resources. Over the last decade, NICE International and Thailand’s Health Intervention and Technology Assessment Program (HITAP) agreed to create partnerships to improve priority setting in LMICs for HTA. Their efforts are well underway in Latin American and Asian countries like Colombia, Vietnam, India, Myanmar and the Philippines [78]. In Africa, some strides have been made in countries like Ghana and South Africa, however, there are still huge gaps including absence of dedicated HTA institutions and limited research capacity [79, 80]. While countries, especially those that have adopted universal health care (UHC) are pushing for HTA to assist them allocate resources appropriately and equitably, as they sustain the UHC programs, studies have suggested that local evidence to inform HTA is limited [81], and further widens the gap between research and policy which is already challenged by low awareness and lack of will among policymakers in the region. We believe our efforts in this review will be very beneficial for policymakers in two facets. First, to feed countries with existing HTA institutions with comprehensive local evidence on interventions that have good value for money as they identify where to invest and guide their HTA efforts. Secondly, our findings will contribute in narrowing the existing knowledge gap on cost-effectiveness on CVD preventive interventions, while highlighting the importance of economic evaluations of interventions as an important guide to resource allocation and priority setting in LMICs with already strained financial resources.

### Recommendations for policy and future research

To bridge the existing knowledge and evidence gap on cost-effectiveness research, and by extension improve the health of populations via provision of cost-effective preventive interventions, experts at the MOH and policy makers should consider; (i) research and capacity building and (ii) the creation of a conducive and enabling environment for the generation of local quality research to inform decisions.

Building research capacity, that is, creation of institutions for economic evaluation and improving technical capacity of local staff via training and workshops will empower local researchers with the skills necessary to generate more local and context-specific evidence to inform policy and decision-making on cost-effective strategies for disease prevention. Encouraging and facilitating partnerships and collaboration between other governments, organizations and researchers within and without the countries are other avenues for capacity building.

Policymakers in the first instance need to develop the political will and interest in cost-effectiveness research and acknowledge its contribution to priority-setting and resource allocation. By so doing, they are likely to more easily understand the funding needs of researchers and organizations, for the generation of the much needed high-quality local evidence. This is particularly important as we note in our review that the evidence-base from LMICs especially the low-income countries is scant. Decisions based on evidence generated from HICs are unlikely to adequately address the needs of these populations due to differences in demographics, intervention effectiveness, variation in healthcare costs and standards of living, cultural differences all likely to affect acceptability, implementation and affordability of interventions.

Taken together, there is a compelling need to link research and policy by improving the interaction between researchers and policymakers via policy meetings, dedicated sessions at conferences where policy makers meet with researchers to discuss evidence, opinions and thus creating opportunities for researchers and their findings to be more actively involved in policy decisions.

In terms of future research, we note that majority (over two-thirds) of studies have focused on pharmacological interventions. Upcoming endeavours should consider looking into non-pharmacological (behavioural and lifestyle) interventions. Secondly, there has been a focus on individual level interventions. Further research on population-level interventions especially those targeting risk factors like salt intake and smoking, and legislative interventions which have in most cases been shown to be very cost-effective and cost-saving are potential areas for focus. For risk factors, most studies have focused on blood pressure, cholesterol, and smoking. We found almost no studies on economic evaluations for reduction in alcohol consumption, physical inactivity, consumption of fruits and vegetables and weight control interventions. These risk factors carry significant burden in LMICs [82], and the limited available interventions for their control highlight important caveats in the literature from the LMICs that need to be explored in future research efforts. Finally, we believe there is need for further work in harmonization and transparence in research analytical methods especially for modelled economic evaluations, as drawing conclusions from such synthesis efforts from studies with largely heterogeneous methods requires a high degree of caution in interpretation of findings, as well as consideration towards transferability and implementation in other settings.

### Strengths and limitations

This systematic review has some limitations that should be discussed. First, limiting our search to only articles in English and French, we might have potentially missed articles in other languages. We however developed a detailed and comprehensive search strategy, accessed multiple databases and grey literature which hopefully should have minimized our missing potential studies. Secondly, a meta-analysis was not done. This is however not surprising for systematic reviews of economic evaluations, owing to the significant heterogeneity in applied methodologies, resources used and evidence on intervention effectiveness. It is important to note that the role of systematic reviews of economic evidence is not just to generate a single summary answer as is generally with systematic reviews of RCTs [83]. The focus here is rather to provide policy/decision makers, clinicians, and stakeholders with information on the variety and quality of available evidence on cost-effectiveness of given interventions, relevant choices and or trade-offs they are likely to contend with, to identify gaps in the literature, and hopefully provide an understanding of the contexts and conditions under which interventions may be cost-effective [83]. Finally, among studies included, 50% were of high quality and further 40% being moderate quality. On the whole, we can therefore have a fair degree of confidence in our findings.

## Conclusions

This systematic review has provided contemporary evidence on the interventions that offer good value for money for the prevention of CVD in LMICs. The bulk of studies focused on pharmacological and other individual-level interventions, which often were found to be cost-effective. Population strategies, though under-represented in the evidence base, are similarly very attractive economically. The available evidence suggests that stemming the CVD epidemic in LMICs would require both individual and population-based strategies to achieve maximal health gains at lowest possible costs. Additionally, there is need for a focus on interventions to address other risk factors like physical inactivity, low fruits and vegetable consumption, alcohol intake and body mass. Decision makers must however not rely exclusively on cost-effectiveness thresholds, but take into account multi-sectoral approaches, and other country and context-specific factors as budget impact, affordability, fairness and implementation as they contemplate which interventions to invest in. Finally, governments in LMICs need to strongly consider strengthening and building research capacity on economic evaluations of interventions, health technology assessment, as well as bridging the gap between research and policy in order to make informed decisions for priority setting towards the allocation of their scarce resources.

## Additional files

**Additional file 1.** PRISMA checklist.

**Additional file 2.** Detailed search strategy.

**Additional file 3.** Drummond quality assessment checklist.

**Additional file 4.** Detailed quality assessment of studies.

## Abbreviations

CVD: cardiovascular disease
GBD: Global Burden of Disease
DALY: disability adjusted life year
QALY: quality adjusted life year
LMIC: low and middle income countries
HIC: high income countries
CEA: cost-effectiveness analysis
CUA: cost utility analysis
CBA: cost benefit analysis
NICE: National Institute for Health and Care Excellence
PRISMA: preferred reporting items for systematic reviews and meta-analysis
MI: myocardial infarction
AF: atrial fibrillation
ACE: angiotensin converting enzyme
