# Supplementary material for: Primary and secondary prevention interventions for cardiovascular disease in low-income and middle-income countries: a systematic review of economic evaluations
Source: Cost Eff Resour Alloc. 2018 Jun 14;16:22. doi: 10.1186/s12962-018-0108-9 (PMC6003072; doi:10.1186/s12962-018-0108-9)
Supplement: Supplementary file 2 — Additional file 2. Detailed search strategy. [file 12962_2018_108_MOESM2_ESM.docx]

1. **For PubMed:**

- **CVD:** (“Cardiovascular disease” OR “Coronary heart disease” OR “ischaemic heart disease” OR “coronary disease” OR “acute coronary syndrome” OR “heart attack” OR “heart disease” OR “atherosclerosis” OR “myocardial infarction” OR “myocardial ischaemia” OR “stroke” OR “cerebrovascular disease” OR “cerebrovascular accident” OR CVA OR “cardiovascular event”) = **850,796 at 3:37:47pm**
- **Prevention:** (“prevention” OR “control” OR “primary prevention” OR “secondary prevention” OR “cardiovascular risk” “risk factor” OR “lifestyle” OR “behaviour” OR diet OR food OR “hypertension” OR “blood pressure” OR “smoking” OR “tobacco” OR alcohol OR “alcohol consumption” OR “physical activity” OR exercise OR salt OR “salt reduction” OR dyslipidaemia OR “lipid lowering” OR cholesterol OR fat OR “intervention” OR “strategies” OR “modification” OR improve OR “address*” OR tax OR “taxation” OR “advertising” OR “counselling” OR “diet advice” OR “health education” OR “patient education”) = **5897052 at 3:37:47pm**
- **Economics:** (“costs and cost analysis” OR “cost-effectiveness” OR “cost-effective” OR “cost-utility” OR “cost benefit” OR “economic evaluation”) = **188602 at 3:38:11pm**
- **Country names:** (“Afghanistan” OR “Albania” OR “Algeria” OR “American Samoa” OR “Angola” OR “Armenia” OR “Azerbaijan” OR “Bangladesh” OR “Belarus” OR “Belize” OR “Benin” OR” “Bhutan” OR “Bolivia” OR “Bosnia and Herzegovina” OR “Botswana” OR “Brazil” OR “Bulgaria” OR “Burkina Faso” OR “Burundi” OR “Cabo Verde” OR “Cambodia” OR “Cameroon” OR “Central African Republic” OR “Chad” OR “China” OR “Colombia” OR “Comoros” OR “Democratic Republic of Congo” OR “Congo” OR “Costa Rica” OR “Cote d’Ivoire” OR “Ivory Coast” OR “Cuba” OR “Djibouti” OR “Dominica” OR “Dominican Republic” OR “Ecuador” OR “Egypt” OR “El Savador” OR “Equatorial Guinea” OR “Eritrea” OR “Ethiopia” OR “Fiji” OR “Gabon” OR “The Gambia” OR “Georgia” OR “Ghana” OR “Grenada” OR “Guatamela” OR “Guinea” OR “Guinea Bissau” OR “Guyana” OR “Haiti” OR “Honduras” OR “India” OR “Indonesia” OR “Iran” OR “Iraq” OR “Jamaica” OR “Jordan” OR “Kazakhastan” OR “Kenya” OR “Kiribati” OR “Democratic People’s Republic of Korea” OR “Kosovo” OR “Kyrgyz Republic” OR “Lao DPR” OR “Lebanon” OR “Lesotho” OR “Liberia” OR “Libya” OR “Macedonia” OR “Madagascar” OR “Malawi” OR “Malaysia” OR “Maldives” OR “Mali” OR “Marshall Islands” OR “Mauritania” OR “Mauritius” OR “Mexico” OR “Micronesia” OR “Moldova” OR “Mongolia” OR “Morocco” OR “Mozambique” OR “Myanmar” OR “Namibia” OR “Nepal” OR “Nicaragua” OR “Niger” OR “Nigeria” OR “Pakistan” OR “Palau” OR “Panama” OR “Papua New Guinea” OR “Paraguay” OR “Peru” OR “Philippines” OR “Romania” OR “Russian Federation” OR “Rwanda” OR “Samoa” OR “Sao Tome and Principe” OR “Senegal” OR “Serbia” OR “Sierra Leonne” OR “Solomon Islands” OR “Somalia” OR “South Africa” OR “South Sudan” OR “Sri Lanka” OR “St Lucia” OR “St Vincent and the Grenadines” OR “Sudan” OR “Suriname” OR “Swaziland” OR “Syrian Arab Republic” OR “Tajikistan” OR “Tanzania” OR “Thailand” OR “Timor-Leste” OR “Togo” OR “Tonga” OR “Tunisia” OR “Turkey” OR “Turkmenistan” OR “Tuvalu” OR “Uganda” OR “Ukraine” OR “Uzbekistan” OR “Vanuatu” OR “Vietnam” OR “West Bank of Gaza” OR “Yemen” OR “Zambia” OR “Zimbabwe” OR Africa OR “sub-Saharan Africa” OR “low and middle income countr*” OR “low income countr*” OR Low OR middle income countr* OR “developing country” OR “underdeveloped country” OR “resource limited”) = **3429579 at 3:38:56pm**
- **Combined search:** Search (((((“Cardiovascular disease” OR “Coronary heart disease” OR “ischaemic heart disease” OR “coronary disease” OR “acute coronary syndrome” OR “heart attack” OR “heart disease” OR “atherosclerosis” OR “myocardial infarction” OR “myocardial ischaemia” OR “stroke” OR “cerebrovascular disease” OR “cerebrovascular accident” OR CVA OR “cardiovascular event”))) AND ((“prevention” OR “control” OR “primary prevention” OR “secondary prevention” OR “cardiovascular risk” “risk factor” OR “lifestyle” OR “behaviour” OR diet OR food OR “hypertension” OR “blood pressure” OR “smoking” OR “tobacco” OR alcohol OR “alcohol consumption” OR “physical activity” OR exercise OR salt OR “salt reduction” OR dyslipidaemia OR “lipid lowering” OR cholesterol OR fat OR “intervention” OR “strategies” OR “modification” OR improve OR “address*” OR tax OR “taxation” OR “advertising” OR “counselling” OR “diet advice” OR “health education” OR “patient education”))) AND ((“costs and cost analysis” OR “cost-effectiveness” OR “cost-effective” OR “cost-utility” OR “cost benefit” OR “economic evaluation”))) AND ((“Afghanistan” OR “Albania” OR “Algeria” OR “American Samoa” OR “Angola” OR “Armenia” OR “Azerbaijan” OR “Bangladesh” OR “Belarus” OR “Belize” OR “Benin” OR” “Bhutan” OR “Bolivia” OR “Bosnia and Herzegovina” OR “Botswana” OR “Brazil” OR “Bulgaria” OR “Burkina Faso” OR “Burundi” OR “Cabo Verde” OR “Cambodia” OR “Cameroon” OR “Central African Republic” OR “Chad” OR “China” OR “Colombia” OR “Comoros” OR “Democratic Republic of Congo” OR “Congo” OR “Costa Rica” OR “Cote d’Ivoire” OR “Ivory Coast” OR “Cuba” OR “Djibouti” OR “Dominica” OR “Dominican Republic” OR “Ecuador” OR “Egypt” OR “El Savador” OR “Equatorial Guinea” OR “Eritrea” OR “Ethiopia” OR “Fiji” OR “Gabon” OR “The Gambia” OR “Georgia” OR “Ghana” OR “Grenada” OR “Guatamela” OR “Guinea” OR “Guinea Bissau” OR “Guyana” OR Haiti” OR “Honduras” OR “India” OR “Indonesia” OR “Iran” OR “Iraq” OR “Jamaica” OR “Jordan” OR “Kazakhastan” OR “Kenya” OR “Kiribati” OR “Democratic People’s Republic of Korea” OR “Kosovo” OR “Kyrgyz Republic” OR “Lao DPR” OR “Lebanon” OR “Lesotho” OR “Liberia” OR “Libya” OR “Macedonia” OR “Madagascar” OR “Malawi” OR “Malaysia” OR “Maldives” OR “Mali” OR “Marshall Islands” OR “Mauritania” OR “Mauritius” OR “Mexico” OR “Micronesia” OR “Moldova” OR “Mongolia” OR “Morocco” OR “Mozambique” OR “Myanmar” OR “Namibia” OR “Nepal” OR “Nicaragua” OR “Niger” OR “Nigeria” OR “Pakistan” OR “Palau” OR “Panama” OR “Papua New Guinea” OR “Paraguay” OR “Peru” OR “Philippines” OR “Romania” OR “Russian Federation” OR “Rwanda” OR “Samoa” OR “Sao Tome and Principe” OR “Senegal” OR “Serbia” OR “Sierra Leonne” OR “Solomon Islands” OR “Somalia” OR “South Africa” OR “South Sudan” OR “Sri Lanka” OR “St Lucia” OR “St Vincent and the Grenadines” OR “Sudan” OR “Suriname” OR “Swaziland” OR “Syrian Arab Republic” OR “Tajikistan” OR “Tanzania” OR “Thailand” OR “Timor-Leste” OR “Togo” OR “Tonga” OR “Tunisia” OR “Turkey” OR “Turkmenistan” OR “Tuvalu” OR “Uganda” OR “Ukraine” OR “Uzbekistan” OR “Vanuatu” OR “Vietnam” OR “West Bank of Gaza” OR “Yemen” OR “Zambia” OR “Zimbabwe” OR Africa OR “sub-Saharan Africa” OR “low and middle income countr*” OR “low income countr*” OR Low OR middle income countr* OR “developing country” OR “underdeveloped country” OR “resource limited”)) = **1385**

2. **For EMBASE**

- **CVD:** 'cardiovascular disease'/exp OR 'cardiovascular disease' OR 'coronary heart disease'/exp OR 'coronary heart disease' OR 'ischaemic heart disease'/exp OR 'ischaemic heart disease' OR 'coronary disease'/exp OR 'coronary disease' OR 'acute coronary syndrome'/exp OR 'acute coronary syndrome' OR 'heart attack'/exp OR 'heart attack' OR 'heart disease'/exp OR 'heart disease' OR 'atherosclerosis'/exp OR 'atherosclerosis' OR 'myocardial infarction'/exp OR 'myocardial infarction' OR 'myocardial ischaemia'/exp OR 'myocardial ischaemia' OR 'stroke'/exp OR 'stroke' OR 'cerebrovascular disease'/exp OR 'cerebrovascular disease' OR 'cerebrovascular accident'/exp OR 'cerebrovascular accident' OR 'cva'/exp OR cva OR 'cardiovascular event'/exp OR 'cardiovascular event' = **3,839,731 at 7:10pm**
- **Prevention/Interventions:** 'prevention'/exp OR 'prevention' OR 'control'/exp OR 'control' OR 'primary prevention'/exp OR 'primary prevention' OR 'secondary prevention'/exp OR 'secondary prevention' OR 'cardiovascular risk'/exp OR 'cardiovascular risk' AND ('risk factor'/exp OR 'risk factor') OR 'lifestyle'/exp OR 'lifestyle' OR 'behaviour'/exp OR 'behaviour' OR 'diet'/exp OR diet OR 'food'/exp OR food OR 'hypertension'/exp OR 'hypertension' OR 'blood pressure'/exp OR 'blood pressure' OR 'smoking'/exp OR 'smoking' OR 'tobacco'/exp OR 'tobacco' OR 'alcohol'/exp OR alcohol OR 'alcohol consumption'/exp OR 'alcohol consumption' OR 'physical activity'/exp OR 'physical activity' OR 'exercise'/exp OR exercise OR 'salt'/exp OR salt OR 'salt reduction' OR 'dyslipidaemia'/exp OR dyslipidaemia OR 'lipid lowering' OR 'cholesterol'/exp OR cholesterol OR 'fat'/exp OR fat OR 'intervention'/exp OR 'intervention' OR 'strategies' OR 'modification'/exp OR 'modification' OR improve OR 'address*' OR 'tax'/exp OR tax OR 'taxation'/exp OR 'taxation' OR 'advertising'/exp OR 'advertising' OR 'counselling'/exp OR 'counselling' OR 'diet advice' OR 'health education'/exp OR 'health education' OR 'patient education'/exp OR 'patient education' = **9,412,356 at 7:10pm**
- **Economics:** 'costs and cost analysis'/exp OR 'costs and cost analysis' OR 'cost-effectiveness'/exp OR 'cost-effectiveness' OR 'cost-effective' OR 'cost-utility'/exp OR 'cost-utility' OR 'cost benefit'/exp OR 'cost benefit' OR 'economic evaluation'/exp OR 'economic evaluation' = **546,421 at 7:11pm**
- **Country names:** 'afghanistan'/exp OR 'afghanistan' OR 'albania'/exp OR 'albania' OR 'algeria'/exp OR 'algeria' OR 'american samoa'/exp OR 'american samoa' OR 'angola'/exp OR 'angola' OR 'armenia'/exp OR 'armenia' OR 'azerbaijan'/exp OR 'azerbaijan' OR 'bangladesh'/exp OR 'bangladesh' OR 'belarus'/exp OR 'belarus' OR 'belize'/exp OR 'belize' OR 'benin'/exp OR 'benin' OR 'bhutan'/exp OR 'bhutan' OR 'bolivia'/exp OR 'bolivia' OR 'bosnia and herzegovina'/exp OR 'bosnia and herzegovina' OR 'botswana'/exp OR 'botswana' OR 'brazil'/exp OR 'brazil' OR 'bulgaria'/exp OR 'bulgaria' OR 'burkina faso'/exp OR 'burkina faso' OR 'burundi'/exp OR 'burundi' OR 'cabo verde' OR 'cambodia'/exp OR 'cambodia' OR 'cameroon'/exp OR 'cameroon' OR 'central african republic'/exp OR 'central african republic' OR 'chad'/exp OR 'chad' OR 'china'/exp OR 'china' OR 'colombia'/exp OR 'colombia' OR 'comoros'/exp OR 'comoros' OR 'democratic republic of congo' OR 'congo'/exp OR 'congo' OR 'costa rica'/exp OR 'costa rica' OR 'cote divoire' OR 'ivory coast'/exp OR 'ivory coast' OR 'cuba'/exp OR 'cuba' OR 'djibouti'/exp OR 'djibouti' OR 'dominica'/exp OR 'dominica' OR 'dominican republic'/exp OR 'dominican republic' OR 'ecuador'/exp OR 'ecuador' OR 'egypt'/exp OR 'egypt' OR 'el savador' OR 'equatorial guinea'/exp OR 'equatorial guinea' OR 'eritrea'/exp OR 'eritrea' OR 'ethiopia'/exp OR 'ethiopia' OR 'fiji'/exp OR 'fiji' OR 'gabon'/exp OR 'gabon' OR 'the gambia'/exp OR 'the gambia' OR 'georgia'/exp OR 'georgia' OR 'ghana'/exp OR 'ghana' OR 'grenada'/exp OR 'grenada' OR 'guatamela' OR 'guinea'/exp OR 'guinea' OR 'guinea bissau'/exp OR 'guinea bissau' OR 'guyana'/exp OR 'guyana' OR 'haiti'/exp OR 'haiti' OR 'honduras'/exp OR 'honduras' OR 'india'/exp OR 'india' OR 'indonesia'/exp OR 'indonesia' OR 'iran'/exp OR 'iran' OR 'iraq'/exp OR 'iraq' OR 'jamaica'/exp OR 'jamaica' OR 'jordan'/exp OR 'jordan' OR 'kazakhastan' OR 'kenya'/exp OR 'kenya' OR 'kiribati'/exp OR 'kiribati' OR 'democratic peoples republic of korea' OR 'kosovo'/exp OR 'kosovo' OR 'kyrgyz republic'/exp OR 'kyrgyz republic' OR 'lao dpr' OR 'lebanon'/exp OR 'lebanon' OR 'lesotho'/exp OR 'lesotho' OR 'liberia'/exp OR 'liberia' OR 'libya'/exp OR 'libya' OR 'macedonia' OR 'madagascar'/exp OR 'madagascar' OR 'malawi'/exp OR 'malawi' OR 'malaysia'/exp OR 'malaysia' OR 'maldives'/exp OR 'maldives' OR 'mali'/exp OR 'mali' OR 'marshall islands'/exp OR 'marshall islands' OR 'mauritania'/exp OR 'mauritania' OR 'mauritius'/exp OR 'mauritius' OR 'mexico'/exp OR 'mexico' OR 'micronesia'/exp OR 'micronesia' OR 'moldova'/exp OR 'moldova' OR 'mongolia'/exp OR 'mongolia' OR 'morocco'/exp OR 'morocco' OR 'mozambique'/exp OR 'mozambique' OR 'myanmar'/exp OR 'myanmar' OR 'namibia'/exp OR 'namibia' OR 'nepal'/exp OR 'nepal' OR 'nicaragua'/exp OR 'nicaragua' OR 'niger'/exp OR 'niger' OR 'nigeria'/exp OR 'nigeria' OR 'pakistan'/exp OR 'pakistan' OR 'palau'/exp OR 'palau' OR 'panama'/exp OR 'panama' OR 'papua new guinea'/exp OR 'papua new guinea' OR 'paraguay'/exp OR 'paraguay' OR 'peru'/exp OR 'peru' OR 'philippines'/exp OR 'philippines' OR 'romania'/exp OR 'romania' OR 'russian federation'/exp OR 'russian federation' OR 'rwanda'/exp OR 'rwanda' OR 'samoa'/exp OR 'samoa' OR 'sao tome and principe'/exp OR 'sao tome and principe' OR 'senegal'/exp OR 'senegal' OR 'serbia'/exp OR 'serbia' OR 'sierra leonne' OR 'solomon islands'/exp OR 'solomon islands' OR 'somalia'/exp OR 'somalia' OR 'south africa'/exp OR 'south africa' OR 'south sudan'/exp OR 'south sudan' OR 'sri lanka'/exp OR 'sri lanka' OR 'st lucia'/exp OR 'st lucia' OR 'st vincent and the grenadines'/exp OR 'st vincent and the grenadines' OR 'sudan'/exp OR 'sudan' OR 'suriname'/exp OR 'suriname' OR 'swaziland'/exp OR 'swaziland' OR 'syrian arab republic'/exp OR 'syrian arab republic' OR 'tajikistan'/exp OR 'tajikistan' OR 'tanzania'/exp OR 'tanzania' OR 'thailand'/exp OR 'thailand' OR 'timor-leste'/exp OR 'timor-leste' OR 'togo'/exp OR 'togo' OR 'tonga'/exp OR 'tonga' OR 'tunisia'/exp OR 'tunisia' OR 'turkey'/exp OR 'turkey' OR 'turkmenistan'/exp OR 'turkmenistan' OR 'tuvalu'/exp OR 'tuvalu' OR 'uganda'/exp OR 'uganda' OR 'ukraine'/exp OR 'ukraine' OR 'uzbekistan'/exp OR 'uzbekistan' OR 'vanuatu'/exp OR 'vanuatu' OR 'vietnam'/exp OR 'vietnam' OR 'west bank of gaza' OR 'yemen'/exp OR 'yemen' OR 'zambia'/exp OR 'zambia' OR 'zimbabwe'/exp OR 'zimbabwe' OR 'africa'/exp OR africa OR 'sub-saharan africa'/exp OR 'sub-saharan africa' OR 'low and middle income countr*' OR 'low income countr*' OR low OR middle AND ('income'/exp OR income) AND countr* OR 'developing country'/exp OR 'developing country' OR 'underdeveloped country'/exp OR 'underdeveloped country' OR 'resource limited' = **117,014 at 7:12pm**

Combined search: 959 entries

**3. For SCOPUS**

( TITLE-ABS-KEY ( "Cardiovascular disease" OR "Coronary heart disease" OR "ischaemic heart disease" OR "coronary disease" OR "acute coronary syndrome" OR "heart attack" OR "heart disease" OR "atherosclerosis" OR "myocardial infarction" OR "myocardial ischaemia" OR "stroke" ) ) AND ( "prevention" OR "control" OR "primary prevention" OR "secondary prevention" OR "cardiovascular risk" "risk factor" OR "lifestyle" OR "behaviour" OR diet OR food OR "hypertension" OR "blood pressure" OR "smoking" OR "tobacco" OR alcohol OR "alcohol consumption" OR "physical activity" OR exercise OR salt OR "salt reduction" OR dyslipidaemia OR "lipid lowering" OR cholesterol OR fat OR "intervention" OR "strategies" OR "modification" OR improve OR "address*" OR tax OR "taxation" OR "advertising" OR "counselling" OR "diet advice" OR "health education" OR "patient education" ) AND ( "costs and cost analysis" OR "cost-effectiveness" OR "cost-effective" OR "cost-utility" OR "cost benefit" OR "economic evaluation" ) AND ( "Afghanistan" OR "Albania" OR "Algeria" OR "American Samoa" OR "Angola" OR "Armenia" OR "Azerbaijan" OR "Bangladesh" OR "Belarus" OR "Belize" OR "Benin" OR "Bhutan" OR "Bolivia" OR "Bosnia and Herzegovina" OR "Botswana" OR "Brazil" OR "Bulgaria" OR "Burkina Faso" OR "Burundi" OR "Cabo Verde" OR "Cambodia" OR "Cameroon" OR "Central African Republic" OR "Chad" OR "China" OR "Colombia" OR "Comoros" OR "Democratic Republic of Congo" OR "Congo" OR "Costa Rica" OR "Cote dIvoire" OR "Ivory Coast" OR "Cuba" OR "Djibouti" OR "Dominica" OR "Dominican Republic" OR "Ecuador" OR "Egypt" OR "El Savador" OR "Equatorial Guinea" OR "Eritrea" OR "Ethiopia" OR "Fiji" OR "Gabon" OR "The Gambia" OR "Georgia" OR "Ghana" OR "Grenada" OR "Guatamela" OR "Guinea" OR "Guinea Bissau" OR "Guyana" OR "Haiti" OR "Honduras" OR "India" OR "Indonesia" OR "Iran" OR "Iraq" OR "Jamaica" OR "Jordan" OR "Kazakhastan" OR "Kenya" OR "Kiribati" OR "Democratic Peoples Republic of Korea" OR "Kosovo" OR "Kyrgyz Republic" OR "Lao DPR" OR "Lebanon" OR "Lesotho" OR "Liberia" OR "Libya" OR "Macedonia" OR "Madagascar" OR "Malawi" OR "Malaysia" OR "Maldives" OR "Mali" OR "Marshall Islands" OR "Mauritania" OR "Mauritius" OR "Mexico" OR "Micronesia" OR "Moldova" OR "Mongolia" OR "Morocco" OR "Mozambique" OR "Myanmar" OR "Namibia" OR "Nepal" OR "Nicaragua" OR "Niger" OR "Nigeria" OR "Pakistan" OR "Palau" OR "Panama" OR "Papua New Guinea" OR "Paraguay" OR "Peru" OR "Philippines" OR "Romania" OR "Russian Federation" OR "Rwanda" OR "Samoa" OR "Sao Tome and Principe" OR "Senegal" OR "Serbia" OR "Sierra Leonne" OR "Solomon Islands" OR "Somalia" OR "South Africa" OR "South Sudan" OR "Sri Lanka" OR "St Lucia" OR "St Vincent and the Grenadines" OR "Sudan" OR "Suriname" OR "Swaziland" OR "Syrian Arab Republic" OR "Tajikistan" OR "Tanzania" OR "Thailand" OR "Timor-Leste" OR "Togo" OR "Tonga" OR "Tunisia" OR "Turkey" OR "Turkmenistan" OR "Tuvalu" OR "Uganda" OR "Ukraine" OR "Uzbekistan" OR "Vanuatu" OR "Vietnam" OR "West Bank of Gaza" OR "Yemen" OR "Zambia" OR "Zimbabwe" OR africa OR "sub-Saharan Africa" OR "low and middle income countr*" OR "low income countr*" OR "middle income countr*" OR "developing country" OR "underdeveloped country" OR "resource limited" ) AND ( EXCLUDE ( AFFILCOUNTRY , "United States" ) OR EXCLUDE ( AFFILCOUNTRY , "United Kingdom" ) OR EXCLUDE ( AFFILCOUNTRY , "Australia" ) OR EXCLUDE ( AFFILCOUNTRY , "Canada" ) OR EXCLUDE ( AFFILCOUNTRY , "Italy" ) OR EXCLUDE ( AFFILCOUNTRY , "Germany" ) OR EXCLUDE ( AFFILCOUNTRY , "Netherlands" ) OR EXCLUDE ( AFFILCOUNTRY , "Spain" ) OR EXCLUDE ( AFFILCOUNTRY , "France" ) OR EXCLUDE ( AFFILCOUNTRY , "Switzerland" ) OR EXCLUDE ( AFFILCOUNTRY , "Sweden" ) OR EXCLUDE ( AFFILCOUNTRY , "Belgium" ) OR EXCLUDE ( AFFILCOUNTRY , "Denmark" ) OR EXCLUDE ( AFFILCOUNTRY , "Japan" ) OR EXCLUDE ( AFFILCOUNTRY , "New Zealand" ) OR EXCLUDE ( AFFILCOUNTRY , "Greece" ) OR EXCLUDE ( AFFILCOUNTRY , "Norway" ) OR EXCLUDE ( AFFILCOUNTRY , "Austria" ) OR EXCLUDE ( AFFILCOUNTRY , "Poland" ) OR EXCLUDE ( AFFILCOUNTRY , "Finland" ) OR EXCLUDE ( AFFILCOUNTRY , "Ireland" ) OR EXCLUDE ( AFFILCOUNTRY , "Singapore" ) OR EXCLUDE ( AFFILCOUNTRY , "Portugal" ) OR EXCLUDE ( AFFILCOUNTRY , "Saudi Arabia" ) OR EXCLUDE ( AFFILCOUNTRY , "Czech Republic" ) OR EXCLUDE ( AFFILCOUNTRY , "Chile" ) OR EXCLUDE ( AFFILCOUNTRY , "Slovakia" ) OR EXCLUDE ( AFFILCOUNTRY , "Croatia" ) OR EXCLUDE ( AFFILCOUNTRY , "Hungary" ) OR EXCLUDE ( AFFILCOUNTRY , "Slovenia" ) OR EXCLUDE ( AFFILCOUNTRY , "United Arab Emirates" ) OR EXCLUDE ( AFFILCOUNTRY , "Qatar" ) OR EXCLUDE ( AFFILCOUNTRY , "Uruguay" ) OR EXCLUDE ( AFFILCOUNTRY , "Iceland" ) OR EXCLUDE ( AFFILCOUNTRY , "Estonia" ) OR EXCLUDE ( AFFILCOUNTRY , "Lithuania" ) OR EXCLUDE ( AFFILCOUNTRY , "Palestine" ) OR EXCLUDE ( AFFILCOUNTRY , "Luxembourg" ) OR EXCLUDE ( AFFILCOUNTRY , "Bahrain" ) OR EXCLUDE ( AFFILCOUNTRY , "Cyprus" ) OR EXCLUDE ( AFFILCOUNTRY , "Kuwait" ) OR EXCLUDE ( AFFILCOUNTRY , "Venezuela" ) OR EXCLUDE ( AFFILCOUNTRY , "Barbados" ) OR EXCLUDE ( AFFILCOUNTRY , "Puerto Rico" ) OR EXCLUDE ( AFFILCOUNTRY , "Seychelles" ) OR EXCLUDE ( AFFILCOUNTRY , "Martinique" ) OR EXCLUDE ( AFFILCOUNTRY , "Anguilla" ) OR EXCLUDE ( AFFILCOUNTRY , "Cook Islands" ) OR EXCLUDE ( AFFILCOUNTRY , "Guadeloupe" ) OR EXCLUDE ( AFFILCOUNTRY , "Guam" ) OR EXCLUDE ( AFFILCOUNTRY , "Undefined" ) ) AND ( EXCLUDE ( SUBJAREA , "AGRI" ) OR EXCLUDE ( SUBJAREA , "ARTS" ) OR EXCLUDE ( SUBJAREA , "BIOC" ) OR EXCLUDE ( SUBJAREA , "CENG" ) OR EXCLUDE ( SUBJAREA , "CHEM" ) OR EXCLUDE ( SUBJAREA , "COMP" ) OR EXCLUDE ( SUBJAREA , "DENT" ) OR EXCLUDE ( SUBJAREA , "EART" ) OR EXCLUDE ( SUBJAREA , "ENER" ) OR EXCLUDE ( SUBJAREA , "PHAR" ) OR EXCLUDE ( SUBJAREA , "ENGI" ) OR EXCLUDE ( SUBJAREA , "IMMU" ) OR EXCLUDE ( SUBJAREA , "ENVI" ) OR EXCLUDE ( SUBJAREA , "SOCI" ) OR EXCLUDE ( SUBJAREA , "MULT" ) OR EXCLUDE ( SUBJAREA , "MATE" ) OR EXCLUDE ( SUBJAREA , "PSYC" ) OR EXCLUDE ( SUBJAREA , "MATH" ) OR EXCLUDE ( SUBJAREA , "PHYS" ) OR EXCLUDE ( SUBJAREA , "VETE" ) ) AND ( EXCLUDE ( LANGUAGE , "Spanish" ) OR EXCLUDE ( LANGUAGE , "Portuguese" ) OR EXCLUDE ( LANGUAGE , "Russian" ) OR EXCLUDE ( LANGUAGE , "Chinese" ) )

Combined search: 931 entries
