# Supplementary material for: Primary and secondary prevention interventions for cardiovascular disease in low-income and middle-income countries: a systematic review of economic evaluations
Source: Cost Eff Resour Alloc. 2018 Jun 14;16:22. doi: 10.1186/s12962-018-0108-9 (PMC6003072; doi:10.1186/s12962-018-0108-9)
Supplement: Supplementary file 3 — Additional file 3. Drummond quality assessment checklist. [file 12962_2018_108_MOESM3_ESM.docx]

| **Item** | | **Yes** | **No** | **Not clear** | **Not appropriate** |
| --- | --- | --- | --- | --- | --- |
| **Study design** | |  |  |  |  |
| 1. | The research question is stated. | ☐ | ☐ | ☐ |  |
| 2. | The economic importance of the research question is stated. | ☐ | ☐ | ☐ |  |
| 3. | The viewpoint(s) of the analysis are clearly stated and justified. | ☐ | ☐ | ☐ |  |
| 4. | The rationale for choosing alternative programmes or interventions compared is stated. | ☐ | ☐ | ☐ |  |
| 5. | The alternatives being compared are clearly described. | ☐ | ☐ | ☐ |  |
| 6. | The form of economic evaluation used is stated. | ☐ | ☐ | ☐ |  |
| 7. | The choice of form of economic evaluation is justified in relation to the questions addressed. | ☐ | ☐ | ☐ |  |
| **Data collection** | |  |  |  |  |
| 8. | The source(s) of effectiveness estimates used are stated. | ☐ | ☐ | ☐ |  |
| 9. | Details of the design and results of effectiveness study are given (if based on a single study). | ☐ | ☐ | ☐ | ☐ |
| 10. | Details of the methods of synthesis or meta-analysis of estimates are given (if based on a synthesis of a number of effectiveness studies). | ☐ | ☐ | ☐ | ☐ |
| 11. | The primary outcome measure(s) for the economic evaluation are clearly stated. | ☐ | ☐ | ☐ |  |
| 12. | Methods to value benefits are stated. | ☐ | ☐ | ☐ | ☐ |
| 13. | Details of the subjects from whom valuations were obtained were given. | ☐ | ☐ | ☐ | ☐ |
| 14. | Productivity changes (if included) are reported separately. | ☐ | ☐ | ☐ | ☐ |
| 15. | The relevance of productivity changes to the study question is discussed. | ☐ | ☐ | ☐ | ☐ |
| 16. | Quantities of resource use are reported separately from their unit costs. | ☐ | ☐ | ☐ |  |
| 17. | Methods for the estimation of quantities and unit costs are described. | ☐ | ☐ | ☐ |  |
| 18. | Currency and price data are recorded. | ☐ | ☐ | ☐ |  |
| 19. | Details of currency of price adjustments for inflation or currency conversion are given. | ☐ | ☐ | ☐ |  |
| 20. | Details of any model used are given. | ☐ | ☐ | ☐ | ☐ |
| 21. | The choice of model used and the key parameters on which it is based are justified. | ☐ | ☐ | ☐ | ☐ |
| **Analysis and interpretation of results** | |  |  |  |  |
| 22. | Time horizon of costs and benefits is stated. | ☐ | ☐ | ☐ | ☐ |
| 23. | The discount rate(s) is stated. | ☐ | ☐ | ☐ | ☐ |
| 24. | The choice of discount rate(s) is justified. | ☐ | ☐ | ☐ | ☐ |
| 25. | An explanation is given if costs and benefits are not discounted. | ☐ | ☐ | ☐ | ☐ |
| 26. | Details of statistical tests and confidence intervals are given for stochastic data. | ☐ | ☐ | ☐ | ☐ |
| 27. | The approach to sensitivity analysis is given. | ☐ | ☐ | ☐ | ☐ |
| 28. | The choice of variables for sensitivity analysis is justified. | ☐ | ☐ | ☐ | ☐ |
| 29. | The ranges over which the variables are varied are justified. | ☐ | ☐ | ☐ | ☐ |
| 30. | Relevant alternatives are compared. | ☐ | ☐ | ☐ | ☐ |
| 31. | Incremental analysis is reported. | ☐ | ☐ | ☐ | ☐ |
| 32. | Major outcomes are presented in a disaggregated as well as aggregated form. | ☐ | ☐ | ☐ |  |
| 33. | The answer to the study question is given. | ☐ | ☐ | ☐ |  |
| 34. | Conclusions follow from the data reported. | ☐ | ☐ | ☐ |  |
| 35. | Conclusions are accompanied by the appropriate caveats. | ☐ | ☐ | ☐ |  |
