# Supplementary material for: Primary and secondary prevention interventions for cardiovascular disease in low-income and middle-income countries: a systematic review of economic evaluations
Source: Cost Eff Resour Alloc. 2018 Jun 14;16:22. doi: 10.1186/s12962-018-0108-9 (PMC6003072; doi:10.1186/s12962-018-0108-9)
Supplement: Supplementary file 4 — Additional file 4. Detailed quality assessment of studies. [file 12962_2018_108_MOESM4_ESM.docx]

**Study design**

**A** = Research question (RQ) is stated

**B** = Economic importance of RQ is stated

**C** = Viewpoint(s) of the analysis clearly stated and justified

**D** = Rationale for choosing alternative programmes/ interventions compared is stated

**E** = Alternatives being compared are clearly described

**F** = Form of economic evaluation used is stated

**G** = Choice of form of economic evaluation is justified in relation to the questions addressed

**Data collection**

**H** = The source(s) of effectiveness used are stated

**I** = Details of the design and results of effectiveness study are given (if based on single study)

**J** = Details of the methods of synthesis or meta-analysis of estimates are given (if based on a synthesis of a number of effectiveness studies)

**K**= The primary outcome measure(s) for the economic evaluation are clearly stated

**L** = Methods to value benefits are stated

**M** = Details of the subjects from whom valuations were obtained were given

**N** = Productivity changes (if included) are reported separately

**O** = The relevance of productivity changes to the study question is discussed

**P** = Quantities of resource use are reported separately from their unit costs

**Q** = Methods for the estimation of quantities and unit costs are described

**R** = Currency and price data are recorded

**S** = Details of currency price adjustments for inflation or currency conversion are given

**T** = Details of any model used are given

**U** = The choice of model used and the key parameters on which it is based are justified

**Analysis and interpretation of results**

**V** = Time horizon of costs and benefits is stated

**W** = The discount rate(s) is stated

**X** = The choice of discount rate(s) is justified

**Y** = An explanation is given if costs and benefits are not discounted

**Z** = Details of statistical tests and confidence intervals are given for stochastic data

**AA** = The approach to sensitivity analysis is given

**AB** = The choice of variables for sensitivity analysis is given

**AC** = The ranges over which the variables are varied are justified

**AD** = Relevant alternatives are compared

**AE** = Incremental analysis is reported

**AF** = Major outcomes are presented in a disaggregated as well as aggregated form

**AG** = The answer to the study question is given

**AH** = Conclusions follow from the data reported

**AI** = Conclusions are accompanied by the appropriate caveats

**AJ** = Total points obtained from Drummond checklist: 1 = Yes, 2 = No, 3 = Not clear, 4 = Not applicable; Yes = 1 point, 2,3 & 4 = 0 point; Total = 35points

**AK** = NICE quality rating [‘++’ = high quality (low risk of bias), ‘+’ = moderate quality (moderate risk of bias), ‘-‘ = low quality (high risk of bias)]
